# HOMA-AD, inflammation, and adipose tissue dysfunction as key drivers of immunometabolic risk in people living with HIV and type 2 diabetes

**DOI:** 10.3389/fendo.2025.1669148

**Published:** 2025-10-07

**Authors:** Elsa J. Anaya-Ambriz, Tania E. Holguín-Aguirre, Paula Catalina Méndez-Ríos, Monserrat Alvarez-Zavala, Luz A. González-Hernández, Jaime F. Andrade-Villanueva, Pedro Martínez Ayala, Rodolfo I. Cabrera-Silva, Karina Sánchez-Reyes

**Affiliations:** ^1^ Programa de Doctorado en Microbiología Médica del Centro Universitario de Ciencias de la Salud de la Universidad de Guadalajara, Guadalajara, Mexico; ^2^ Unidad de VIH, Hospital Civil de Guadalajara “Fray Antonio Alcalde”, Guadalajara, Mexico; ^3^ Programa de Doctorado en Ciencias en Biología Molecular en Medicina del Centro Universitario de Ciencias de la Salud de la Universidad de Guadalajara, Guadalajara, Mexico; ^4^ Departamento de Clínicas Médicas, Instituto de Investigación en Inmunodeficiencias y VIH, Centro Universitario de Ciencias de la Salud, Universidad de Guadalajara, Guadalajara, Mexico

**Keywords:** HOMA-AD, insulin-resistance, chronic-inflammation, HIV, T2D

## Abstract

**Background:**

The increased life expectancy of people living with HIV (PLWHIV), due to the effectiveness of antiretroviral therapy, has been associated with a higher incidence of metabolic disorders such as dyslipidemia, insulin resistance, and adipose tissue redistribution. It has been demonstrated that the secretion of adipokines, particularly adiponectin, a key hormone in the regulation of inflammation and metabolism, is altered by these changes. This study it is intended to evaluate the HOMA-AD index and its correlations with metabolic, inflammatory, and obesity-related parameters.

**Methods:**

Eighty participants were divided into five groups (PLWHIV, PLWHIV+preT2D, PLWHIV+T2D, PWT2D, and Controls). Clinical history, anthropometric data, and blood samples were collected to assess biochemical parameters. Adiponectin, hs-CRP, IL-6, IL-18, and IL-8 levels were quantified by ELISA. HOMA2-IR, HOMA2-%B, HOMA2-%S, HOMA-AD, and surrogate IR indices (TyG, TyG-BMI, TyG-WHtR, METS-IR, QUICKI) were calculated. Adiposity indices (VAI, DAI) and inflammatory markers (TG/HDL-C, NEU/HDL-C, MON/HDL, PLT/HDL, NLR, PLR) were also evaluated. Analyses were performed using IBM SPSS, GraphPad Prism, and RStudio.

**Results:**

The discrimination of T2D in PLWHIV was effectively achieved by the indices HOMA-AD, TyG-WHtR, QUICKI, and METS-IR, with AUC values reaching up to 0.9. Moderate correlations were identified between HOMA-AD and METS-IR (R = 0.58), TyG-WHtR (R = 0.53), QUICKI (R = -0.90), DAI (R = 0.45), and VAI (R = 0.44), as well as inflammatory markers hs-CRP (R = 0.30), IL-6 (R = 0.25), and IL-18 (R = 0.27). A cutoff point of HOMA-AD >1 was associated with a significantly increased risk for T2D in PLWHIV (OR = 15.4; 95% CI: 2.79–79.5), x (OR = 1.97), and non-HIV T2D populations (OR = 9.53). These results highlight the importance of HOMA-AD and inflammatory markers in glycemic risk stratification.

**Conclusions:**

Our study demonstrates that the HOMA-AD index improves T2D detection in PLWHIV, likely due to its strong association with insulin resistance, systemic inflammation, and adiposity. It emerges as a promising tool to evaluate metabolic and inflammatory status in this population.

## 1 Introduction

An increasing incidence of non-communicable diseases (NCDs) has been observed globally among people living with HIV (PLWHIV), attributable in part to the prolonged longevity and efficacy of antiretroviral therapy (ART) (1). The introduction of highly effective ART has led to a rising burden of comorbidities, particularly metabolic disorders. These include dyslipidemia, insulin resistance (IR), altered glucose metabolism, and changes in adipose tissue (AT) distribution (2, 3). These metabolic disturbances affect AT distribution, including adipocyte size and lipid content. They also influence the number and type of immune cells in AT and the secretion of adipokines (3).

Evidence indicates that HIV generates persistent immune activation and chronic inflammation, leading to increased metabolic complications and organ damage. ART-related metabolic effects, together with common risk factors such as smoking, alcohol use, diet, physical activity, and age contribute to metabolic disorders development (1).

In PLWHIV it has been reported a prevalence of IR or T2D of 46%, with an incidence of 1.2/100 patients-years (4), and in relation to gender through HOMA-IR the rate varies little with 57.5% for women and 47.3% for men (5). In HIV context, IR prevalence seem to be predominant among patients on ART, however this condition appears to be independent of the ART, but if associated with obesity (6).

IR is associated with a wide spectrum of metabolic conditions such as T2D, metabolic syndrome (MetS) and its complications such as cardiovascular diseases (1).The proportion of people starting integrase inhibitor-based therapies (INSTIs) has increased since 2007. Patients starting INSTIs based therapies, compared those on other regimens, show greater weight gain and a 31% higher risk of developing new-onset diabetes and hyperglycemia, especially during the first 6 months. However, these findings are also replicated in people whose regimens are optimized toward one containing integrase inhibitors (7–9).

Adiponectin (AD) is one of the main circulating adipokines, which also play a role in regulating metabolism and immune function. AT contains adipocytes and immune cells that produce proinflammatory cytokines and respond to immunological signals. Inflammation is associated with metabolic stress resulting from excessive adipocyte growth due to overnutrition and external factors such as lipopolysaccharides, these stimuli activate local cytokine production, leading to AT remodeling and IR (10).

Some adipokines, such as AD, reduce the risk of cardiovascular disease by regulating glucose and lipid metabolism through reducing inflammation and oxidative stress (11). The glucose regulation generated by AD suggests that it is an insulin-sensitizing adipokine that may converge with insulin in some signaling pathways (11).

As an insulin-sensitizing adipokine, AD mainly acts in the liver and skeletal muscle. First, it increases lipoprotein lipase activity in white AT, enhancing triglyceride uptake and reducing triglyceride storage in the liver and muscle. Second, it promotes fatty acid oxidation in skeletal muscle. Both effects can reduce ectopic lipid storage in the liver and muscle, reversing lipid-induced IR (11). AD is the most abundant protein secreted by AT and exhibits potent anti-inflammatory properties. In contrast to other adipokines, AD levels decrease in the presence of proinflammatory factors, such as TNF-α, IL-6, reactive oxygen species, and hypoxia in animal models of obesity and IR (10, 12).

Currently, there is a growing focus on rapidly and easily identifying IR. This approach avoids costly, invasive, and technically complex methods, such as the euglycemic-hyperinsulinemic clamp (13). In this context, several surrogate indices have been developed that incorporate, in addition to glucose and insulin, anthropometric parameters (e.g, BMI and waist circumference) and components of the lipid profile (e.g., triglycerides and HDL-C) (14). These approaches enable a more comprehensive assessment of metabolic status, offering advantages in clinical practicality, accessibility, and acceptable correlation with the clamp method (15).

Inflammation and IR are closely linked in particular in context of obesity and T2D. Some indices are also useful for evaluating systemic inflammation. Ratios such as TG/HDL-C, NEU/HDL-C, MON/HDL-C, PLT/HDL-C, and the neutrophil-to-lymphocyte ratio (NLR) and platelet-to-lymphocyte ratio (PLR), are linked not only to metabolic disturbances but also to inflammatory biomarkers, including IL-6, IL-8, IL-18, and high-sensitivity C-reactive protein (hs-CRP) (16, 17). Their functionality positions these indices as accessible and clinically relevant tools that may enable an integrated evaluation of metabolic and inflammatory status, contributing to improved patient stratification and monitoring (18, 19).

One of the surrogate indices that integrates both metabolic and inflammatory components is the HOMA-AD (Homeostasis Model Assessment–Adiponectin), first introduced in 2014. HOMA-AD modifies the original HOMA-IR formula by adding circulating AD levels, a key adipokine with anti-inflammatory and insulin-sensitizing effects (20). HOMA-AD has been primarily used to assess IR in both adult and pediatric populations with obesity, MetS, or T2D, demonstrating stronger correlations with the euglycemic-hyperinsulinemic clamp method compared to traditional indices (21, 22).

HOMA-AD is also linked to systemic inflammatory markers, including IL-6 and hs-CRP (23, 24), and to other composite indices of IR and inflammation. These findings suggest that HOMA-AD is a valuable biomarker for simultaneously assessment of metabolic dysfunction and low-grade inflammation in diverse clinical contexts.

Recent studies indicate that HOMA-AD may surpass traditional indices such as HOMA1-IR by incorporating adiponectin, thereby reflecting both IR and low-grade inflammation. In adults, HOMA-AD demonstrated stronger correlations with hyperglycemic clamp measurements and higher AUCs for identifying IR and MetS, with an optimal cutoff of 0.95, highlighting its potential utility in clinical and research settings (22).

HOMA-AD has demonstrated clinically relevant cut-off values for detecting IR and MetS. In adults, the index showed an AUC of 0.846 with an optimal cut-off of ≥6.26, sensitivity of 68.14%, and specificity of 85.33%. In obese adolescents, HOMA-AD cut-offs were ≥0.37 for IR (sensitivity 74.17%, specificity 84.85%) and ≥0.43 for MetS (sensitivity 71.52%, specificity 59.60%). These thresholds may guide early detection, risk stratification, and intervention strategies, including among PLWHIV (25, 26).

However, despite its clinical relevance, the utility of HOMA-AD in PLWHIV has not been thoroughly explored, and to our knowledge, this is the first study to evaluate HOMA-AD in this population. Because chronic inflammation, dysregulated adipokine secretion, and metabolic dysfunction converge in PLWHIV, the assessment of HOMA-AD may offer valuable insights into the mechanisms underlying the development of T2D in this population. Therefore, the aim of this study is to investigate the associations between HOMA-AD, surrogate indices of IR and inflammation, and the risk of T2D in PLWHIV.

## 2 Materials and methods

### 2.1 Study population and ethical statement

This cross-sectional study was conducted at the HIV and Immunodeficiencies Research Institute (InIVIH) of the University of Guadalajara, in Guadalajara, Jalisco, Mexico. The study adhered to the ethical principles for medical research involving human subjects as outlined in the 1975 Declaration of Helsinki (revised in Brazil, 2013) and was approved by the Research Ethics Committee of the Hospital Civil de Guadalajara “Fray Antonio Alcalde” (HCG-FAA), registration number 138/22; HCG/CEI/0884/22 and as well as by the Research, Ethics, and Biosafety Committees of the University Center of Health Sciences at the University of Guadalajara, registration number 20-39; CI-07720; CUCS/CINV/0220/20. Written informed consent was obtained from all participants prior to their inclusion in the study.

### 2.2 Study design and patient selection

A total of 80 adult participants were included, distributed into five distinct groups of 16 participants each. PLWHIV were recruited of the HCG-FAA HIV Unit. Specifically, for PLWHIV the inclusion criteria were adults aged 18 to <65 years, on antiretroviral therapy (ART), with at least 1.5 years of undetectable HIV viral load, and CD4^+^ T-cell count >300 cells/μL. Additionally, all eligible participants were classified according to their glycemic condition determined by the guidelines of the American Diabetes Association (ADA) (27): PLWHIV in normoglycemia (PLWHIV), PLWHIV with prediabetes (PLWHIV+preT2D), PLWHIV with type 2 diabetes (PLWHIV+T2D); in addition, from the general population, people living with type 2 diabetes (PLWT2D) and control group in normoglycemia, were included.

### 2.3 Serological status and clinical characteristics

Viral load and CD4+ T-cell count were performed with Roche AmpliPrep/COBAS^®^ TaqMan^®^ HIV-1 Test platform and FACS Calibur platform (BD, Indianapolis); respectively. Clinical characteristics of PLWHIV were validated in the electronic medical record (SMART HIV database) of the HCG-FAA HIV Unit. In the case of PLWT2D and control group, glycemic conditions were validated through the evaluation of fasting glucose and insulin, performed in the HCG-FAA Central Laboratory.

### 2.4 Data collection and laboratory assessment

A complete medical history was obtained to gather demographic data, as well as non-pathological and pathological antecedents. Subsequently, anthropometric measurements were taken to determine weight, height, body mass index (BMI), waist circumference, hip circumference, and waist-to-hip ratio (WHR). Briefly, waist circumference was measured at the midpoint between the lower margin of the last palpable rib and the iliac crest, and hip circumference at the widest portion of the buttocks, according to WHO recommendations (28).

Blood samples were collected from fasting patients, and sent to the Central Laboratory of HCG-FAA for determination of hematological, lipid, and glycemic profiles; or processed to obtain serum. Briefly, the whole blood was left to rest for 20 min and then centrifuged at 1800 rpm/7 min to obtain serum, which was aliquoted and stored at -80 °C until use.

### 2.5 Measurement of AD and inflammatory cytokines

The determinations of AD and cytokines were performed using commercial kits. AD.

was quantified using the Human Adiponectin ELISA Kit (Cat. No. KHP0041, Invitrogen, Brazil). Interleukin-6 (IL-6) was measured with the Human IL-6 ELISA Kit High Sensitivity (Cat. No. ab46042, Abcam, Cambridge). For hs-CRP, the CRP, HS (C-Reactive Protein) ELISA Kit (Cat. No. EIA-3954, DRG, Germany) was used. IL-8 and IL-18 were measured using the LEGENDplex™ Human Inflammation Panel 1, capture beads B2 and B6 (Cat. No. 740809, BioLegend, Inc., San Diego, CA, USA).

Determinations of AD and cytokines were performed strictly according to the manufacturers’ instructions, using validated standard curves for data interpolation, ensuring measurement consistency and reliability. For ELISAs assays, the absorbances were read using the Biotek Synergy H1 microplate reader at the specific wavelength indicated for each analyte. The obtained optical densities (OD) were analyzed and converted into concentrations, expressed in pg/mL or ng/mL, as per the manufacturer’s instructions for each kit used. For flow cytometry assay, data were acquired in an Attune Acoustic Focusing Cytometer (Life Technologies, Carlsbad, CA, USA). More than 2,000 events for each analyte were acquired. The files were analyzed using LEGENDplex™ QOGNIT virtual software (BioLegend, Inc., San Diego, CA, USA). Values are expressed in pg/mL.

### 2.6 Calculation of HOMA-AD and surrogate indices of insulin resistance

Each of the indices was calculated using their respective equations, which are presented below. Additionally, the evaluation of the Homeostasis Model Assessment 2 for IR (HOMA-2IR), the Homeostasis Model Assessment 2 for β-cell Function (HOMA-2B), and HOMA2-S was performed using the HOMA calculator version 2.2.3 provided by the Radcliffe Department of Medicine, University of Oxford.


$$\text{HOMA}-\text{AD}=\frac{\left[\text{fasting glucose }(\text{mmol}/\text{L})\text{ x Fasting insulin}(\text{mU}/\text{L})\right]}{\left[22.5\text{ x AD }(\text{g}/\text{mL})\right]}$$

$\text{TyG}=\text{ln}(\frac{\left[\text{fasting TG }(\text{mg}/\text{dL})\text{ x Fasting glucose }(\text{mg}/\text{dL})\right]}{2})$ .


TyG−BMI=TyG x BMI

$$\text{TyG}-\text{WHtR}=\text{TyG x }(\frac{\text{Waist }(\text{cm})}{\text{Height }(\text{m})}÷100)$$

$$\text{METS}-\text{IR}=\frac{\text{ln }([2\text{ x fasting glucose }(\text{mg}/\text{dL})]+\text{TG}(\text{mg}/\text{dL}))\text{xBMI}}{\text{ln}(\text{HDL}-\text{C})}$$

$$\text{QUICKI}=\frac{1}{\left[\log(\text{Fasting insulin }(\text{µ}\frac{\text{U}}{\text{mL}}))+\log(\text{fasting glucose }(\frac{\text{mg}}{\text{dL}}))\right]}$$

$$\text{Women}\;\text{VAI}=(\frac{\text{Waist }(\text{cm})}{36.58+(1.89\text{*BMI})})*(\frac{\text{TG }(\frac{\text{mmol}}{\text{L}})}{0.81})*(\frac{1.52}{\text{HDL}-\text{C }(\frac{\text{mmol}}{\text{L}})})$$

$$\text{Men}\;\text{VAI}=(\frac{\text{Waist }(\text{cm})}{39.68+(1.88\text{*BMI})})*(\frac{\text{TG }(\frac{\text{mmol}}{\text{L}})}{1.03})*(\frac{1.31}{\text{HDL}-\text{C }(\frac{\text{mmol}}{\text{L}})})$$

$$\text{Men}\;\text{DAI}=(\frac{\text{Waist }(\text{cm})}{22.79+(2.68\text{*BMI})})*(\frac{\text{TG }(\frac{\text{mmol}}{\text{L}})}{1.37})*(\frac{1.19}{\text{HDL}-\text{C }(\frac{\text{mmol}}{\text{L}})})$$

$$\text{Women}\;\text{DAI}=(\frac{\text{Waist }(\text{cm})}{24.02+(2.37\text{*BMI})})*(\frac{\text{TG }(\frac{\text{mmol}}{\text{L}})}{1.32})*(\frac{1.43}{\text{HDL}-\text{C }(\frac{\text{mmol}}{\text{L}})})$$

$$\text{TG}/\text{HDL}-\text{C}=\frac{\text{TG }(\text{mg}/\text{dL})}{\text{HDL}-\text{C }(\text{mg}/\text{dL})}$$

$$\text{NEU}/\text{HDL}-\text{C}=\frac{\text{Neutrophil count}}{\text{HDL}-\text{C }(\text{mg}/\text{dL})}$$

$$\text{MON}/\text{HDL}-\text{C}=\frac{\text{Monocyte count}}{\text{HDL}-\text{C }(\text{mg}/\text{dL})}$$

$$\text{PLT}/\text{HDL}-\text{C}=\frac{\text{Platelet count}}{\text{HDL}-\text{C }(\text{mg}/\text{dL})}$$

$$\text{NLR}=\frac{\text{Neutrophil count}}{\text{Lymphocyte count}}$$

$$\text{PLR}=\frac{\text{Platelet count}}{\text{Lymphocyte count}}$$

### 2.7 Statistical analysis

A descriptive analysis was conducted to characterize the study population, using measures of central tendency (mean or median) and dispersion (standard deviation or interquartile ranges) for quantitative variables. For qualitative variables, results were expressed as frequencies and percentages. Comparisons between groups were performed using ANOVA or the Kruskal-Wallis test, with *post hoc* analysis conducted using Tukey or Dunn tests, depending on the data distribution. For qualitative variables, the chi-square test (χ²) was employed. Additionally, receiver operating characteristic (ROC) curve analyses were performed for each surrogate index of IR, and optimal cutoff points were determined using the Youden index. Correlations between HOMA-AD and the selected variables were assessed using Spearman’s rank correlation test. Odds ratios (ORs) were estimated through contingency tables, applying either Fisher’s exact test or the chi-square test (χ²), depending on the expected cell counts. All statistical analyses were performed using IBM SPSS Statistics (version 29.0.2.0) and GraphPad Prism (version 10.3.0), Statistical significance level was set at p < 0.05.

## 3 Results

A total of 80 subjects were included and distributed across five study groups, with 16 participants to each group based on their serological status for HIV and glycemic condition. Sociodemographic characteristics, healthy habits and anthropometric profile were primarily described in **Table 1**.

**Table 1:** Sociodemographic characteristics, healthy habits and anthropometric profile of study groups.

	PLWHIV (n=16)	PLWHIV+preT2D (n=16)	PLWHIV+T2D (n=16)	PLWT2D (n=16)	Control (n=16)	P value
Sociodemographic and healthy habits						
Age (years)	36.5	49	50	56.5	44.5	
	(22 – 63)	(25-64)	(31-56)	(30-68)	(25-71)	**0.01**
Gender %						
Male	12 (75)	16 (100)	14 (87.5)	9 (56.25)	8 (50)	
Female	4 (25)	-	2 (12.5)	7 (43.75)	8 (50)	**0.001**
Smoking%						
Negative	12 (75)	11 (68.75)	14 (87.5)	13 (81.25)	12 (75)	
positive	4 (25)	5 (31.25)	2 (12.5)	3 (18.75)	4 (25)	0.76
AU %						
Negative	6 (37.5)	7 (43.75)	4 (25)	7 (43.75)	5 (31.25)	
Low risk	6 (37.5)	9 (56.25)	9 (56.25)	8 (50)	9 (56.25)	
High risk	4 (25)	-	3 (18.75)	1 (6.25)	2 (12.5)	0.54
PA level %						
Null	7 (43.75)	8 (50)	11 (68.75)	7 (43.75)	8 (50)	
Mild	5 (31.25)	3 (18.75)	2 (12.5)	5 (31.25)	4 (25)	
Moderate	4 (25)	4 (25)	3 (18.75)	4 (25)	4 (25)	
Intense	-	1 (6.25)	-	-	-	0.84
Anthropometric profile						
Weight (Kg)	69.3 ± 9	73.4 ± 15.6	84.9 ± 17.9	80 ± 12.8	76 ± 15.6	**0.031**
Height (m)	1.71 (1.5 – 1.8)	1.7 (1.6 – 1.9)	1.71 (1.5 – 1.8)	1.69 (1.5 – 1.8)	1.67 (1.5 – 1.9)	0.7
BMI (Kg/m²)	24.5 (19 – 32)	24.7 (19 – 40)	28.4 (21 – 39)	29.4 (21 – 37)	26.2 (20 – 40)	**0.007**
Waist (cm)	85 ± 8.7	90 ± 15.3	96.5 ± 7.1	95.9 ± 11	89 ± 12.7	**0.007**
Hip (cm)	97 (85 – 120)	101 (85 – 109)	101 (91 – 113)	103 (90 – 119)	102 (80 – 123)	0.077
WHR	0.9 (0.6 – 1)	0.9 (0.8 – 1.3)	1 (0.8 – 1.1)	0.9 (0.8 – 1)	0.9 (0.7 – 0.9)	**0.003**

AU, Alcohol Use; PA, Physical Activity; BMI, Body Mass Index; WHR, Waist-to-Hip Ratio. The table presents measures of central tendency and dispersion. Quantitative variables with a normal distribution are reported as mean with standard deviation, while quantitative variables with a non-normal distribution are presented as median with minimum and maximum values. Qualitative variables are reported as frequency and percentage. Statistical tests used include ANOVA and Kruskal-Wallis for quantitative variables, and Chi-square (χ²) for qualitative variables. A p-value of <0.05 was considered statistically significant.The values in bold indicates statistical significance.

A statistically significant difference in age was identified, with higher median values observed in the PLWHIV+T2D (50 years) and PLWT2D (56.5 years) groups compared to the remaining groups (p = 0.01). Regarding sex distribution, a statistically significant difference was observed (p = 0.001), a predominance of the male gender was observed in all groups except for the control group, in which an equal distribution of males and females (50% each) was recorded.

With respect to smoking status, no statistically significant differences were detected among groups (p = 0.76); however, the highest proportion of current smokers was noted in the PLWHIV+preT2D group (31.25%). Concerning alcohol use, no statistically significant differences were found (p = 0.54). The PLWHIV group exhibited the highest frequency of high-risk alcohol consumption (25%), whereas the PLWT2D group presented the lowest (6.25%). Regarding physical activity, 51.2% of the total sample reported no engagement in any physical activity, while the remaining 48.8% participated in moderate to intense physical activity. Although no statistically significant differences were observed among the groups (p = 0.84), only three individuals (18.75%) in the PLWHIV+T2D group reported moderate activity, and a single participant (6.25%) in the PLWHIV group reported intense physical activity.

In relation to anthropometric profiles, statistically significant differences were found in body weight (p = 0.031), with the highest values recorded in the PLWHIV+T2D group (84.9 ± 17.9 kg). Significant differences were also observed in body mass index (BMI) (p = 0.007), waist circumference (p = 0.007), and waist-to-hip ratio (WHR) (p = 0.003), all of which were elevated in the groups with type 2 diabetes. No significant differences were found in height (p = 0.70) or hip circumference (p = 0.077) (**Table 1** **).**

IR contributes to the metabolic alterations observed in HIV-infected patients; additionally, AD is a hormone that plays an important role in insulin sensitivity, a condition that should be primarily detected in PLWHIV, since these individuals are at high risk for metabolic diseases such as T2D. In this regard, it was deemed essential to describe their clinical characteristics to adequately contextualize the metabolic and immunological findings. Important variables related to HIV infection were assessed and are shown in **Table 2**, included time since HIV diagnosis, current ART regimen, viral load, current CD4^+^ T cell count, and CD4^+^ T nadir as a marker of immune reconstitution.

**Table 2:** Clinical and treatment characteristics of PLWHIV.

	PLWHIV (n=16)	PLWHIV+preT2D (n=16)	PLWHIV+T2D (n=16)	P value
HIV diagnostic time	3 (1 – 19)	11 (1 – 37)	9.5 (2 – 23)	**0.008**
Current ART Biktarvy^®^ Atripla^®^ Truvada^®^ + ATV/r	16 (100) - -	15 (93.8) 1 (6.2) -	15 (93.8) - 1 (6.2)	NA
Time on ART (years)	3 (1 – 19)	10.5 (1 – 20)	9.5 (2 – 23)	**0.009**
Viral load (copies/mL)	31 (20 – 40)	20 (19 – 40)	34.5 (19 – 46)	0.28
CD4^+^ T cell (cells/μL)	535 (250 – 1,310)	591 (301 – 1,117)	586 (328 – 1151)	0.73
Nadir CD4^+^ T cell (cells/μL)	237 (15 – 829)	194 (11 – 778)	404 (10 – 2,181)	0.08

DX, Diagnosis; ART, Antiretroviral Therapy; ATV/r, Atazanavir/ritonavir. Quantitative variables are reported as median with minimum and maximum values. Qualitative variables are reported as frequency and percentage. Statistical tests used include Kruskal-Wallis for quantitative variables, and Chi-square (χ²) for qualitative variables. A p-value <0.05 was considered statistically significant.The values in bold indicates statistical significance.

Statistically significant differences were identified in HIV diagnosis time (p = 0.008), with higher median values observed in the PLWHIV+preT2D (11 years) and PLWHIV+T2D (9.5 years) groups, compared to the PLWHIV group (3 years). A similar pattern was observed for time on ART, which also differed significantly among the groups (p = 0.009).

Biktarvy^®^ was the predominant regimen across all groups, being used by 100% of participants in the PLWHIV group, and by 93.8% in both the PLWHIV+preT2D and PLWHIV+T2D groups. The remaining participants were treated with Atripla^®^ (n = 1, PLWHIV+preT2D) or Truvada^®^ + ATV/r (n = 1, PLWHIV+T2D). No statistically significant differences were observed in viral load levels (p = 0.28), with medians ranging between 20 and 34.5 copies/mL, consistent with virologic suppression. Similarly, no significant differences were found in current CD4^+^ T cell counts (p = 0.73), with median values ranging from 535 to 591 cells/μL. Although differences in CD4^+^ nadir were not statistically significant (p = 0.08), a trend toward higher nadir values was noted in the PLWHIV+T2D group, suggesting a more favorable immune reconstitution profile (**Table 2** **).**

To comprehensively contextualize the participants’ metabolic profile, a detailed evaluation of hematological, lipid, and glycemic parameters was conducted across the five study groups. These variables provided relevant insight into the general systemic status and cardiometabolic risk.

Statistically significant differences were identified in hemoglobin levels (p = 0.0002), with lower values observed in the PLWT2D group (13.5 ± 1.7 g/dL) compared to the other groups. No significant differences were found in platelet count, total leukocytes, neutrophils, lymphocytes, or monocytes (p > 0.05 in all cases). Regarding the lipid profile, HDL cholesterol (HDL-C) levels were significantly lower in the PLWHIV+T2D group (median: 32 mg/dL) compared to the other groups (p = 0.001). No statistically significant differences were observed in total cholesterol, LDL-C, VLDL, or triglyceride levels (p > 0.05). In contrast, the glycemic profile revealed marked differences. The PLWHIV+T2D and PLWT2D groups exhibited significantly higher fasting glucose and glycated hemoglobin levels (p < 0.0001), consistent with their diabetic condition. Likewise, insulin concentrations were significantly elevated in these two groups, with the highest levels observed in the PLWT2D group (p = 0.007), indicating a progressive increase in insulin levels associated with dysglycemia (**Table 3** **).**

**Table 3:** Serum lipid profiles and glycemic profile of study groups.

	PLWHIV (n=16)	PLWHIV+preT2D (n=16)	PLWHIV+T2D (n=16)	PLWT2D (n=16)	Control (n=16)	P value
Hematological profile						
Hb (g/dL)	15.3 ± 1.6	15.6 ± 0.8	15.5 ± 1.6	13.5 ± 1.7	15 ± 0.9	**0.0002**
PLT (10³/μL)	229 (140 – 305)	214 (127 – 262)	227 (142 – 410)	221 (57 – 304)	233 (163 – 330)	0.73
WBC (10³/μL)	5.2 (4 – 9)	6.3 (4 – 14)	6.3 (4 – 12)	6.2 (3 – 11)	5.9 (4 – 10)	0.38
NEU (10³/μL)	2.6 (1 – 7)	3.3 (1 – 10)	3 (1 – 6)	3 (2 – 6)	3 (0 – 7)	0.32
LYM (10³/μL)	2 (1 – 3)	2 (0.9 – 3)	2 (1 – 5)	2 (0.6 – 4)	2 (1 – 3)	0.42
MONO (10³/μL)	0.5 (0.3 – 0.7)	0.4 (0.2 – 1)	0.4 (0.3 – 1)	0.3 (0.1 – 1)	0.4 (0.2 – 0.7)	0.09
Lipid profile						
TC (mg/dL)	176 (116 – 368)	175.5 (123 – 266)	172 (127 – 250)	162.5 (101 – 252)	196.5 (112 –260)	0.39
LDL-C (mg/dL)	108 ± 35.7	105 ± 27.3	99 ± 34.3	98 ± 36	107 ± 35.8	0.91
HDL-C (mg/dL)	42.5 (27 – 66)	42.5 (27 –67)	32 (26 – 45)	42.6 (24 – 58)	49 (24 – 114)	**0.001**
VLDL (mg/dL)	22 (9 – 74)	21 (11 – 67)	32.5 (15 – 109)	28.5 (15 – 60)	22.5 (11 – 95)	0.091
TG (mg/dL)	111.5 (45 – 368)	105 (56 – 333)	179.5 (76 – 546)	143.5 (67 – 302)	121.5 (53 – 476)	0.10
Glycemic profile						
Glu (mg/dL)	83.5 (69 – 101)	85 (72 – 100)	138 (80 – 281)	148 (79 – 254)	82.5 (70 – 99)	**<0.0001**
HbA1c (%)	5.4 ± 0.3	5.7 ± 0.2	8.3 ± 2.3	7.6 ± 1.7	5.7 ± 0.3	**<0.0001**
Ins (µu/mL)	10.95 (3 – 44)	9.46 (3 – 45)	17.8 (6 – 39)	25.7 (6 – 48)	9.5 (5 – 34)	**0.007**

Hb, Hemoglobin; PLT, Platelets; WBC, White Blood Cells; NEU, Neutrophils; LYM, Lymphocytes; MONO, Monocytes; TC, Total Cholesterol; LDL-C, Low-Density Lipoprotein Cholesterol; HDL-C, High-Density Lipoprotein Cholesterol; VLDL, Very Low-Density Lipoprotein; TG, Triglycerides; Glu, Glucose; HbA1c, Glycated Hemoglobin; Ins: Insulin. The table presents measures of central tendency and dispersion. Quantitative variables with a normal distribution are reported as mean with standard deviation, while quantitative variables with a non-normal distribution are presented as median with minimum and maximum values. Statistical tests used include ANOVA and Kruskal-Wallis. A p-value of <0.05 was considered statistically significant.The values in bold indicates statistical significance.

To complement the characterization of the participants’ systemic status, five inflammatory and immunometabolic biomarkers were evaluated across the five study groups: hs-CRP, IL-6, IL-18, IL-8 and AD. These biomarkers provide insights into subclinical inflammation and immunometabolic dysfunction, which are relevant to the pathophysiology of cardiometabolic diseases, particularly in people living with HIV.

Statistically significant differences were observed in AD concentrations (p = 0.011), with lower median levels detected in the PLWHIV+T2D group (3 µg/mL; minimum–maximum: 2.3–6.8), compared to the PLWHIV+preT2D group (5 µg/mL; minimum–maximum: 4.4–13.4). This progressive decline may be associated with the metabolic deterioration characteristic of T2D. Similarly, hs-CRP levels differed significantly between groups (p = 0.005), with markedly higher values in the PLWHIV+T2D group (median: 18.5 mg/L; minimum–maximum: 2.5–170), indicating a more pronounced proinflammatory state. Although no statistically significant differences were found in IL-6 or IL-8 concentrations (p = 0.288 and p = 0.214; respectively), a trend toward higher IL-6 levels was noted in the PLWHIV+T2D group. In contrast, IL-18 concentrations showed statistically significant differences among the groups (p = 0.032), with the highest values recorded in the PLWHIV+T2D group (median: 377 pg/mL; minimum–maximum: 150–995), these results highlight the evident enhanced inflammatory response in PLWHIV+T2D group (**Table 4** **).**

**Table 4:** Inflammatory and inmunometabolic profile, surrogate indices of IR, indices of visceral adiposity and dysfunctional adiposity and markers of systemic inflammation and cardiovascular risk across study groups. .

	PLWHIV (n=16)	PLWHIV+preT2D (n=16)	PLWHIV+T2D (n=16)	PLWT2D (n=16)	Control (n=16)	P value
Inflammatory and immunometabolic profile						
AD (µg/mL)	4 (2.7 – 15)	5 (4.4 – 13.4)	3 (2.3 – 7)	4 (3 – 6)	4 (3 – 11)	**0.011**
hs-CRP (mg/L)	2.5 (0.3 – 70)	1.8 (0.7 – 105)	18.5 (2.5 – 170)	4 (1 – 114)	2 (0.7 – 45)	**0.005**
IL-6 (pg/mL)	2 (1.2 – 4)	1.8 (0.2 – 6)	2.5 (0.7 – 16.7)	2 (0.3 – 10.9)	1.7 (0.4 – 5)	0.288
IL-18 (pg/mL)	348 (159 – 796)	226 (71 – 696)	377 (150 – 995)	263 (100 – 532)	193 (92 – 548)	**0.032**
IL-8 (pg/mL)	104 (5 – 211)	68 (36 – 355)	93 (28 – 184)	63 (17 – 164)	71 (15 – 175)	0.214
Surrogate indices of insulin resistance						
HOMA2-IR	1.4 (0.4 – 5.5)	1.3 (0.4 – 5.3)	2.6 (0.9 – 5.5)	3.9 (0.8 – 7)	1.3 (0.6 – 4.3)	**0.001**
HOMA2-%B	124 (44 – 283)	120 (41 – 385)	60 (10 – 274)	101 (19 – 279)	146 (73 – 332)	**0.034**
HOMA2-%S	71.3 (18 – 270)	83 (5.3 – 248)	38 (19 – 118)	26 (4 – 132)	83 (23 – 162)	**0.001**
HOMA-AD	0.5 (0 – 4.1)	0.3 (0.1 – 1.7)	1.7 (0.7 – 6.3)	3 (0.2 – 7.3)	0.7 (0.1 – 3.1)	**<0.0001**
TyG	8.4 (7.5 – 9.7)	8.4 (7.7 – 9.6)	9.6 (8.2 – 11.2)	9.4 (8.3 – 10.3)	8.6 (7.7 – 9.9)	**0.0002**
TyG*BMI	210 ± 38	218 ± 49	279 ± 50	269 ± 37	231 ± 44	**<0.0001**
TyG-WHtR	4.3 ± 0.8	4.5 ± 0.8	5.4 ± 0.6	5.4 ± 0.7	4.6 ± 0.8	**<0.0001**
METS-IR	45.6 ± 7.8	39.3 ± 9.2	52.6 ± 9.5	48.1 ± 7.1	40.4 ± 8.2	**<0.0001**
Quicki	0.35 ± 0.05	0.34 ± 0.04	0.29 ± 0.03	0.29 ± 0.04	0.34 ± 0.03	**<0.0001**
Indices of visceral and dysfunctional adiposity.						
VAI	1.5 (0.4 – 5)	1.5 (0.7 – 4.7)	3.4 (1.2 – 11.5)	2.6 (0.8 – 6.6)	1.6 (0.4–12.6)	**0.006**
DAI	1 (0.3 – 3.3)	0.95 (0.5 – 2.9)	2.2 (0.7 – 7.3)	1.7 (0.5 – 4.2)	0.95 (0.2 – 8.4)	**0.010**
markers of systemic inflammation and cardiovascular risk						
TG/HDL-c ratio	2.7 (0.9 – 9.7)	2.4 (1.4 – 7.9)	5.2 (2.2 – 18.8)	4.2 (1.4 – 10)	2.3 (0.6–19.4)	**0.008**
Neu/HDL-C	1.3 (0.6 – 1.8)	1.3 (0.7 – 2.2)	1.6 (0.7 – 2.5)	1.6 (0.8 – 2.5)	1.1 (0.6 – 2.5)	0.053
Mon/HDL-C	0.010 (0.01–0.02)	0.011 (0.01 – 0.03)	0.013 (0.01 – 0.04)	0.009 (0 – 0.02)	0.008 (0 – 0.02)	**0.046**
Plt/HDL-C	5.7 (2.6 – 8.8)	5.1 (3.7 – 8.3)	6.7 (4.3 – 14.1)	6.1 (0.6 – 9.1)	4.9 (1.9 – 9.1)	**0.021**
NLR	1.6 (0.5 – 5.1)	1.9 (0.5 – 4.1)	1.3 (0.4 – 3.5)	1.7 (0.8 – 3.2)	1.9 (1.1 – 2.8)	0.31
PLR	118 (54 – 193)	108 (53 – 170)	95.3 (58 – 192)	116 (14 – 367)	123 (75 – 191)	0.24

AD, Adiponectin; hs-CRP, High-sensitivity C-reactive protein; HOMA-AD, Homeostasis Model Assessment-Adiponectin; TyG,Triglyceride-Glucose Index; TyG*BMI, Triglyceride-Glucose Index multiplied by Body Mass Index; TyG-WHtR, Triglyceride-Glucose Index multiplied by Waist-to-Height Ratio; METS-IR, Metabolic Score for Insulin Resistance; QUICKI, Quantitative Insulin Sensitivity Check Index; VAI, Visceral Adiposity Index; DAI, Dysfunctional Adipose Index; TG/HDL-C, Triglyceride to HDL-Cholesterol Ratio; NEU/HDL-C, Neutrophil to HDL-Cholesterol Ratio; MON/HDL-C, Monocyte to HDL-Cholesterol Ratio; PLT/HDL-C, Platelet to HDL-Cholesterol Ratio; NLR, Neutrophil to Lymphocyte Ratio; PLR, Platelet to Lymphocyte Ratio. The table presents measures of central tendency and dispersion. Quantitative variables with a normal distribution are reported as mean with standard deviation, while quantitative variables with a non-normal distribution are presented as median with minimum and maximum values. Statistical tests used include ANOVA and Kruskal-Wallis. A p-value of <0.05 was considered statistically significant.The values in bold indicates statistical significance.

Determining IR in PLWHIV is key to ensuring adequate clinical management of the progression of metabolic diseases. Thus, to complements the metabolic profile analysis, various surrogate indices of IR were evaluated across the five study groups. These indices allow for indirect estimation of insulin sensitivity and beta-cell function, serving as valuable tools to characterize dysglycemia in different clinical contexts. Additionally, the advantage is that these incorporate variables beyond glucose and insulin, including lipid, glycemic, anthropometric profiles as well as AD an insulin-sensitizing hormone, with a key role in glucose metabolism.

Statistically significant differences were observed in the HOMA2-IR index (p = 0.001), with the highest values recorded in the PLWHIV+T2D group (median: 2.6, minimum–maximum: 0.9–5.5) and the PLWT2D group (median: 3.9, minimum–maximum: 0.8–7), indicating greater IR in these diabetic subgroups. A significant reduction in beta-cell function (HOMA2-%B) was found (p = 0.034) in the PLWHIV+T2D group (median: 60, minimum–maximum: 10–274), compared to controls and other groups. Interestingly, PLWHIV-preT2D showed marked beta-cell function, a sign of a compensatory response to glycemic imbalance. Insulin sensitivity (HOMA2-%S) also showed significant differences (p = 0.001), being notably lower in the PLWHIV+T2D (median: 38, minimum–maximum: 19–118) and PLWT2D groups (median: 26, minimum–maximum: 4–132).

Other indices reflecting insulin resistance, such as HOMA-AD, TyG, TyG*BMI, TyG-WHtR, METS-IR, and QUICKI, presented highly significant differences (p < 0.0001), evidencing progressive metabolic deterioration in the diabetic groups, especially in PLWHIV+T2D and PLWT2D groups. For example, median HOMA-AD values of 1.7 and 3 were observed in the PLWHIV+T2D and PLWT2D groups, respectively, substantially higher than in the other groups. (**Table 4**).

Adiposity and AT dysfunction indices were also evaluated in the five study groups with the purpose of exploring alterations in body composition and adipose metabolism, which are key factors in the pathophysiology of IR and cardiometabolic diseases.

Statistically significant differences were observed in the Visceral Adiposity Index (VAI) among groups (p = 0.006), with higher median values reported in the PLWHIV+T2D group (3.4, minimum–maximum: 1.2–11.5), followed by the PLWT2D group (2.6, minimum–maximum: 0.8–6.6), reflecting greater impairment in visceral AT function and distribution in these diabetic subgroups. Similarly, the AT dysfunction index (DAI) also showed significant differences (p = 0.010), with elevated median levels found in the PLWHIV+T2D group (2.2, minimum–maximum: 0.7–7.3) and the PLWT2D group (1.7, minimum–maximum: 0.5–4.2), compared to the non-diabetic groups, as well as PLWT2D a dysfunction in the AT was evident in PLHIV+T2D, which is characterized by a proinflammatory adipokine secretion pattern, inflammatory cell infiltration particularly in intra-abdominal fat, and represents a potential link to metabolic and cardiovascular diseases. (**Table 4**).

In this sense, it was in our interest to evaluate markers of systemic inflammation and cardiovascular risk across the five study groups to identify possible variations associated with metabolic and inflammatory status. The TG/HDL ratio was also significantly elevated in the PLWHIV+T2D group (median: 5.2; minimum–maximum: 2.2–18.8), reflecting a more atherogenic lipid profile (p = 0.008). On the other hand, the ratio NEU/HDL-C showed a trend toward statistical significance (p = 0.053), with higher median values observed in the PLWHIV+T2D and PLWT2D groups (both 1.6, range: 0.7–2.5 and 0.8–2.5; respectively), suggesting increased systemic inflammation and cardiovascular risk in these diabetic groups. Significant differences were identified in the MON/HDL-C ratio (p = 0.046), with the highest median levels found in the PLWHIV+T2D group (0.013, range: 0.01–0.04), followed by PLWHIV+preT2D (0.011, range: 0.01–0.03), indicating increased monocyte-mediated inflammatory activity in relation to the lipid profile. The PLT/HDL-C ratio also showed significant differences between groups (p = 0.021), with the PLWHIV+T2D group presenting the highest median value (6.7, range: 4.3–14.1), which may reflect enhanced platelet activation, and a pro-thrombotic state associated with cardiovascular risk. As systemic inflammatory markers the NLR and PLR was evaluated, no statistically significant differences were found (p = 0.31, p = 0.56; respectively), although variations in median values were observed without a clear pattern across groups (**Table 4**).

To more clearly illustrate the differences observed among the study groups, scatter plots were constructed for selected variables representative of key metabolic and inflammatory domains. These variables were chosen based on their clinical relevance and either statistical significance or biologically meaningful trends observed in the primary analysis.

**Figure 1:**
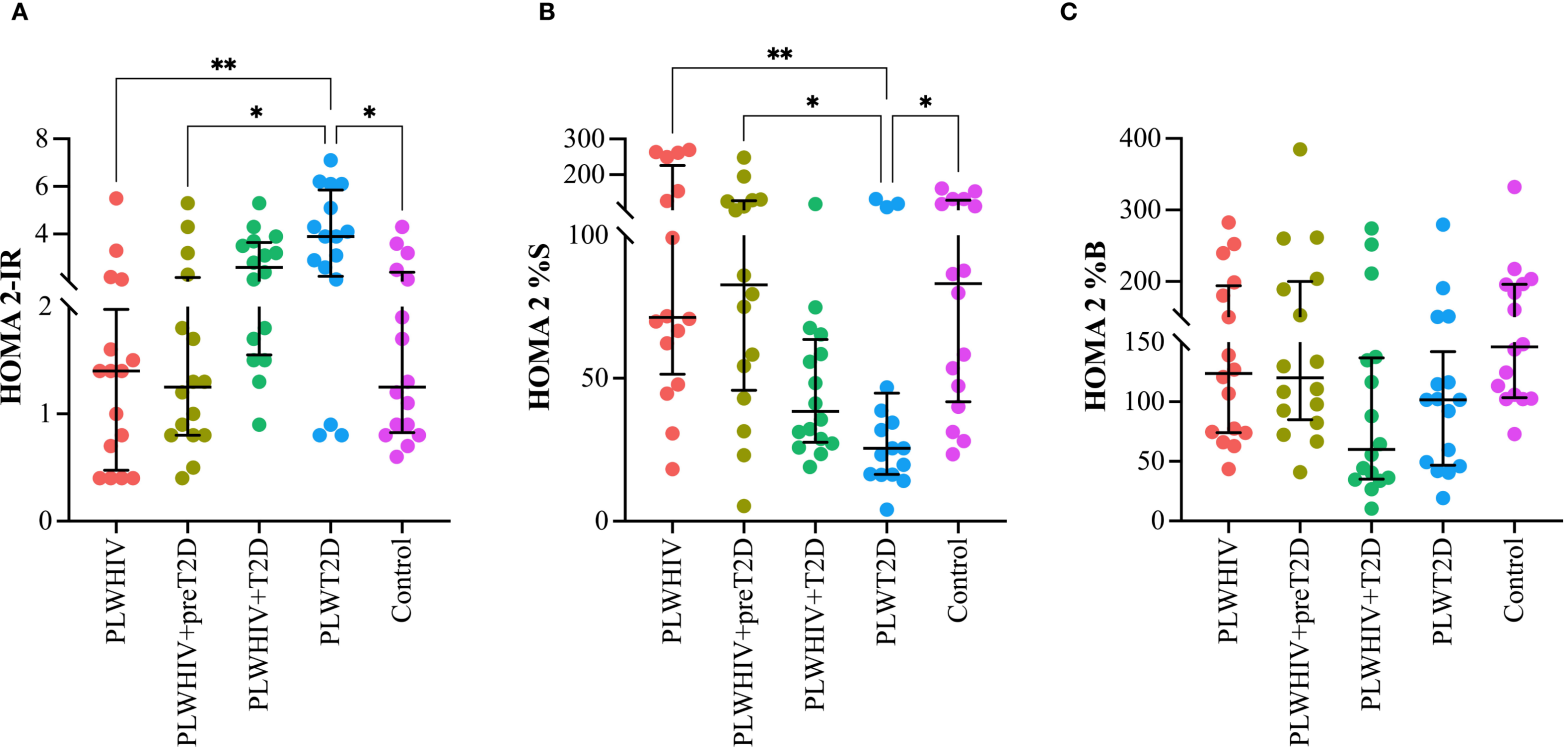
Indices of insulin resistance, insulin sensitivity, and beta-cell function in the study groups. Scatter plots depicting **(A)** HOMA-2IR (insulin resistance), **(B)** HOMA-S (insulin sensitivity), and **(C)** HOMA-B (beta-cell function) across the study groups. Median values and interquartile ranges are shown. Statistical comparisons were performed using the Kruskal-Wallis test followed by Dunn’s *post hoc* test. *p < 0.05, **p < 0.01.

A significant increase in the HOMA 2-IR index was observed in the PLWT2D group compared to the PLWHIV (p= 0.005), PLWHIV+preT2D (p= 0.002), and control group (p= 0.04), suggesting greater IR in the presence of T2D. Conversely, the HOMA 2%S index was found to be significantly decreased in the PLWT2D group relative to all other groups, except for PLWHIV+T2D, indicating reduced insulin sensitivity in individuals with diabetes. Regarding the HOMA2-%B index, no statistically significant differences were detected among the study groups. However, a trend toward lower values was noted in both the PLWHIV+T2D and PLWT2D groups when compared to controls, potentially indicating pancreatic beta-cell dysfunction in individuals with T2D, regardless of HIV serostatus. (**Figure 1** **).**

**Figure 2:**
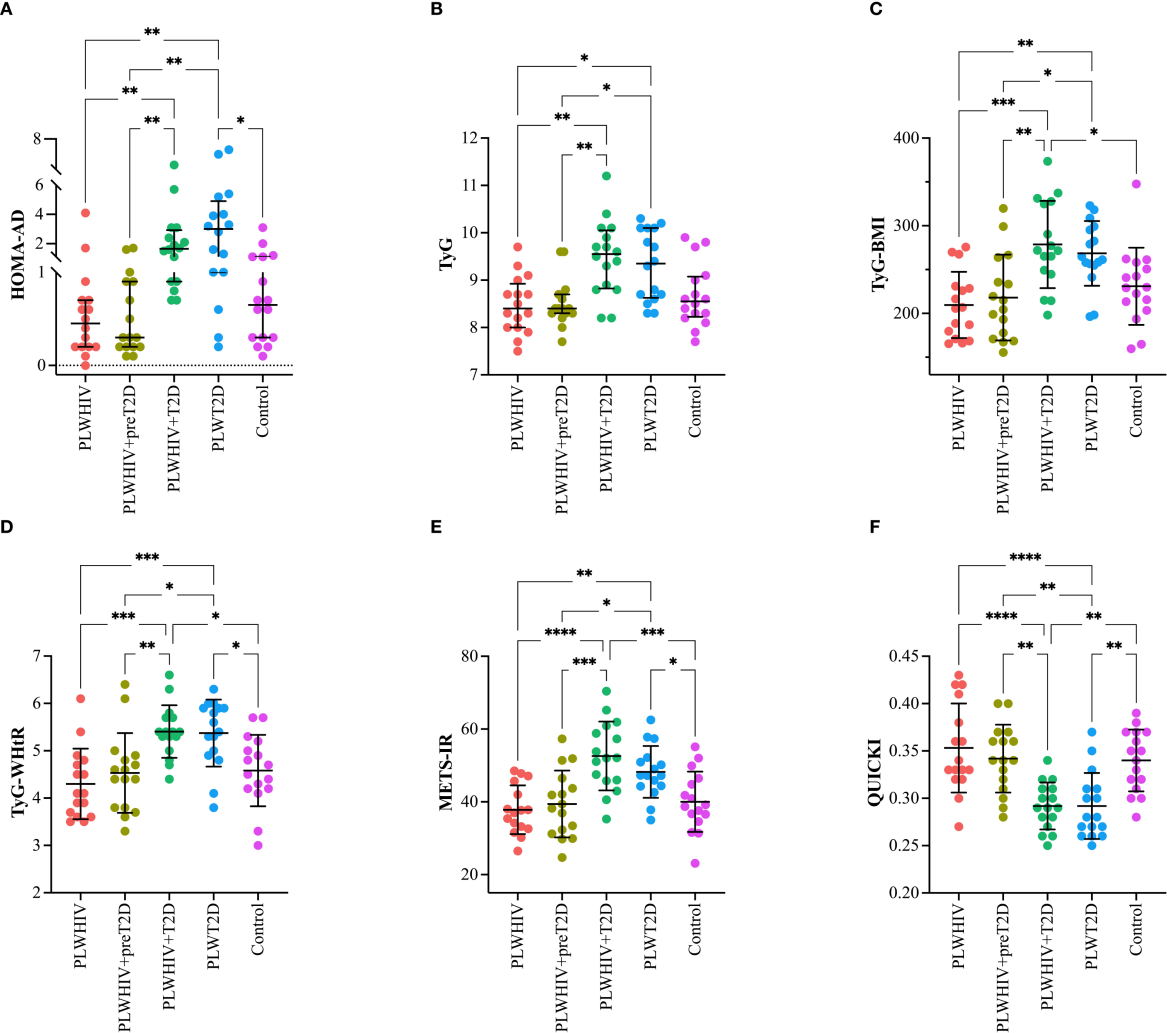
Surrogate indices of IR in the study groups. Scatter plots depicting **(A)** HOMA-AD (adiponectin-adjusted insulin resistance), **(B)** TyG (triglyceride-glucose index), **(C)** TyG-BMI (triglyceride-glucose index adjusted for body mass index), **(D)** TyG-WHtR (triglyceride-glucose index adjusted for waist-to-height ratio), **(E)** METS-IR (metabolic score for insulin resistance), and **(F)** QUICKI (quantitative insulin sensitivity check index) in the study groups. Median values and interquartile ranges are shown. Statistical comparisons were performed using the Kruskal-Wallis test followed by Dunn’s *post hoc* test. *p < 0.05, **p < 0.01, ***p < 0.001, ****p<0.0001.

The indices HOMA-AD, TyG, TyG-BMI, TyG-WHtR, METS-IR, and QUICKI were assessed in the different study groups. The HOMA-AD index, a marker of IR adjusted for AD, was found to be significantly elevated in the PLWHIV+T2D and PLWT2D groups. Statistically significant differences were identified when comparing the PLWHIV+T2D group with PLWHIV (p = 0.003) and with PLWHIV+preT2D (p = 0.0027). Additionally, the PLWT2D group exhibited significantly higher values compared to PLWHIV (p = 0.0013), PLWHIV+preT2D (p = 0.0012), and the control group (p = 0.0357) (**Figure 2A**). The TyG index, a surrogate marker of IR based on triglyceride and glucose levels, was found to be significantly elevated in the PLWHIV+T2D and PLWT2D groups compared to PLWHIV (p = 0.005 and p = 0.021, respectively) and PLWHIV+preT2D (p = 0.009 and p = 0.030; respectively) (**Figure 2B**). The TyG*BMI and TyG-WHtR indices, which incorporate anthropometric measures to assess IR, were found to follow a pattern similar to that of the TyG index, with significant increases observed in the PLWHIV+T2D and PLWT2D groups, and additionally for both indices, PLWHIV+T2D group shows significant differences with the control group (p < 0.02 and p = 0.01; respectively). (**Figures 2C, D**).

The METS-IR index, a metabolic marker of insulin resistance, was found to be significantly elevated in the PLWHIV+T2D and PLWT2D groups. When compared to the other groups, significant differences were observed relative to PLWHIV (p < 0.0001 and p = 0.005, respectively), PLWHIV+preT2D (p = 0.0002 and p = 0.027, respectively), and the control group (p = 0.0004 and p = 0.46, respectively) (**Figure 2E**). Finally, the QUICKI index, a marker of insulin sensitivity, was found to be significantly reduced in the PLWHIV+T2D and PLWT2D groups compared to the other groups, indicating decreased insulin sensitivity in individuals with T2D (**Figure 2F**).

**Figure 3:**
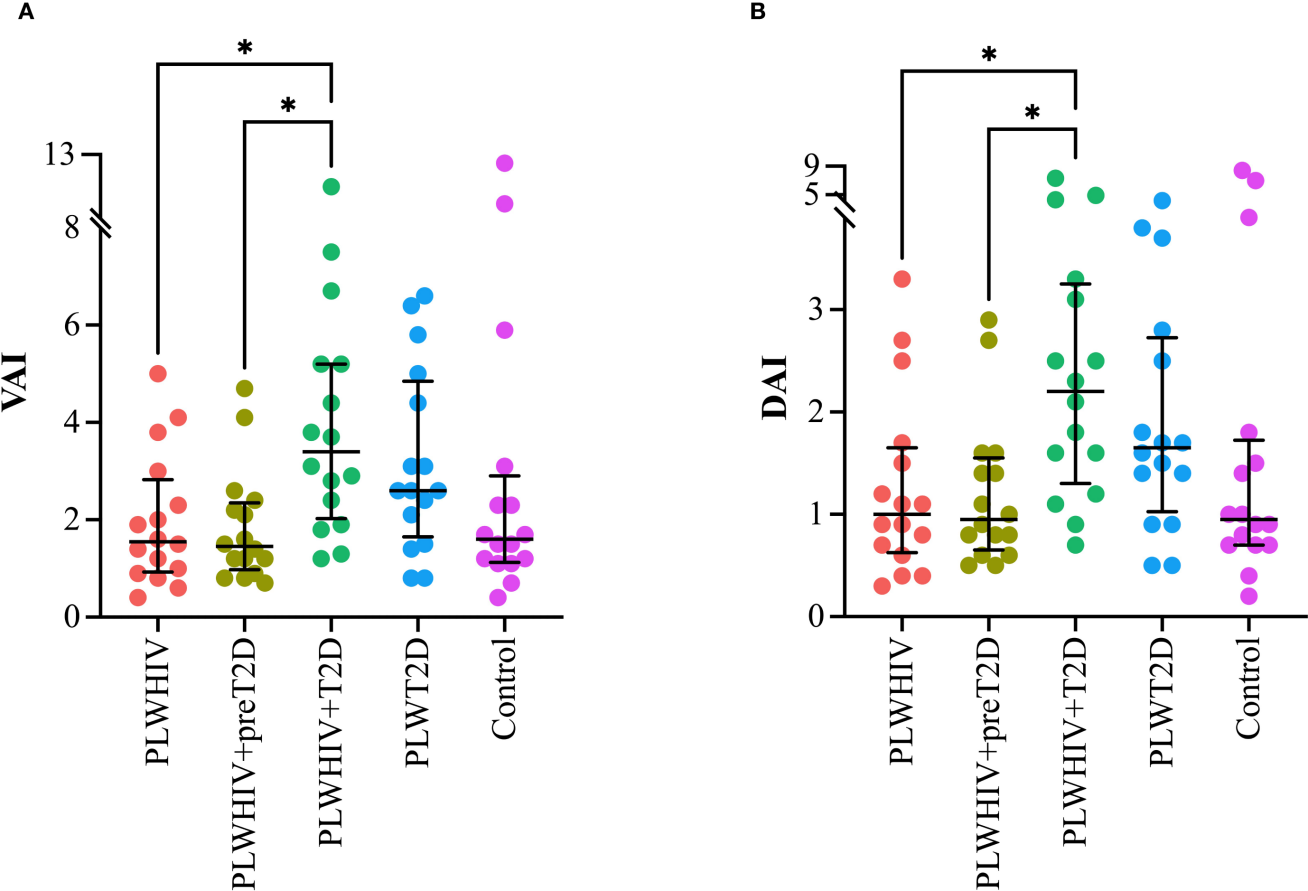
Adiposity and adipose dysfunction indices. Scatter plots depicting. **(A)** VAI (Visceral Adiposity Index), **(B)** DAI (Dysfunctional Adipose Index). Median values and interquartile ranges are shown. Statistical comparisons were performed using the Kruskal-Wallis test followed by Dunn’s *post hoc* test. *p < 0.05.

Visceral Adiposity Index (VAI) and the Dysfunctional AT Index (DAI) were found to be significantly elevated in the PLWHIV+T2D group. For VAI, statistically significant differences were observed when compared to the PLWHIV (p = 0.042) and PLWHIV+preT2D groups (p = 0.022). Similarly, the DAI followed the same pattern, with significant differences relative to the PLWHIV (p = 0.047) and PLWHIV+preT2D groups (p = 0.038) (**Figure 3**).

**Figure 4:**
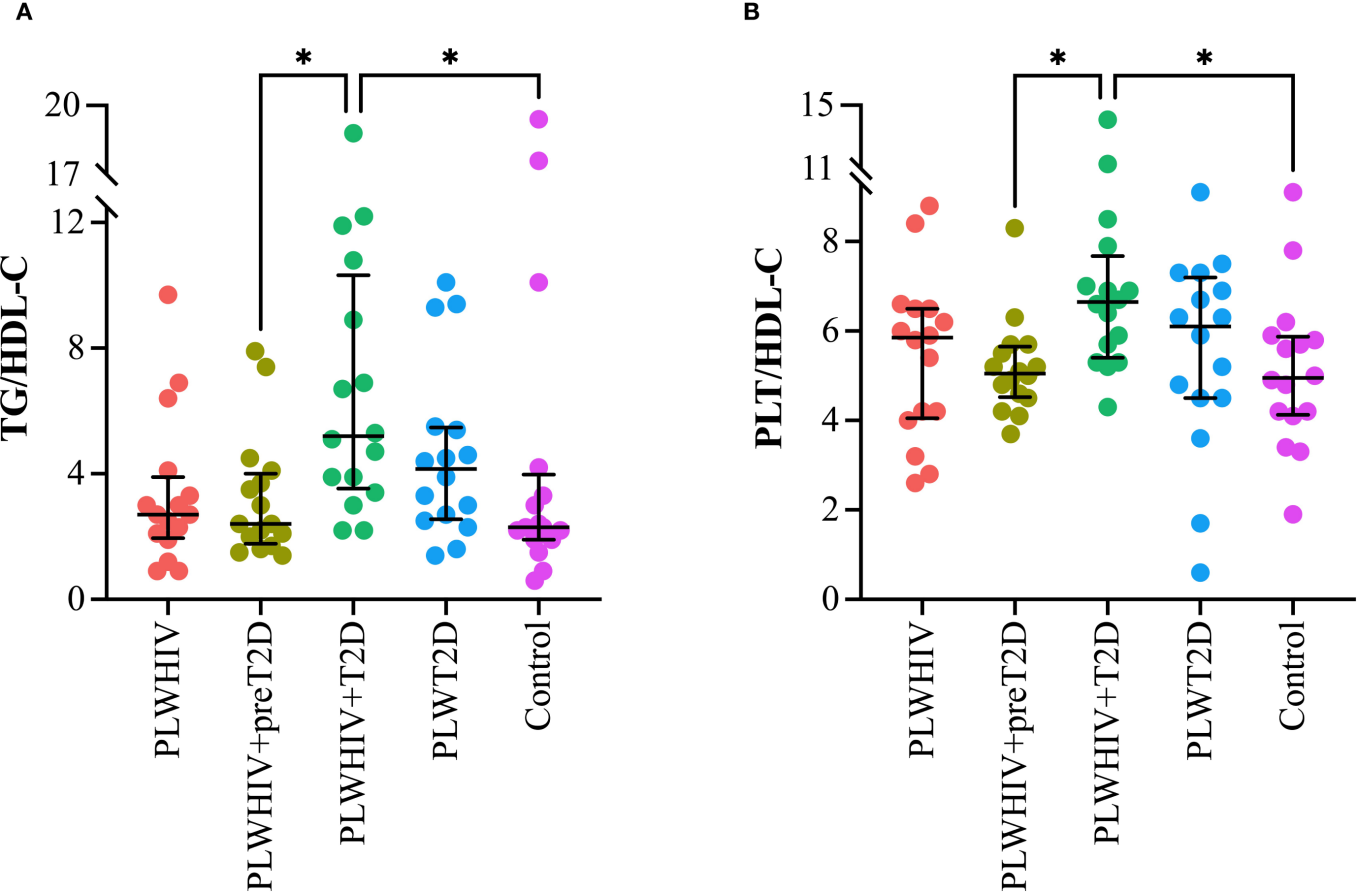
Markers of systemic inflammation and cardiovascular risk. Scatter plots depicting. **(A)** TG/HDL-C: Triglyceride to HDL-Cholesterol Ratio, **(B)** PLT/HDL-C: Platelet to HDL-Cholesterol Ratio. Median values and interquartile ranges are shown. Statistical comparisons were performed using the Kruskal-Wallis test followed by Dunn’s *post hoc* test. *p < 0.05.

Markers of inflammation and cardiovascular risk, assessed through the TG/HDL-C and PLT/HDL-C ratios, were found to be significantly elevated in the PLWHIV+T2D group. Both ratios exhibited a similar pattern, with statistically significant differences observed when compared to the PLWHIV+preT2D group (p = 0.03) and the control group (p = 0.04) (**Figure 4** **).**

**Figure 5:**
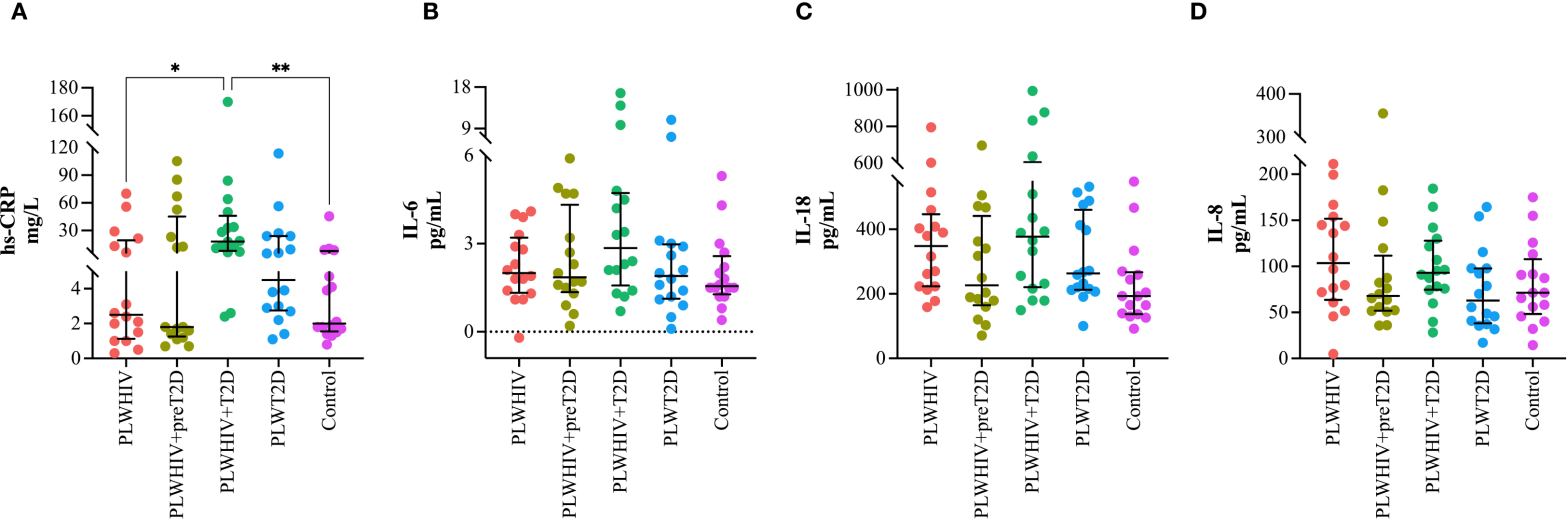
Inflammatory cytokines. Scatter plots depicting. **(A)** hs-CRP, **(B)** IL-6, **(C)** IL-18, **(D)** IL-8. Median values and interquartile ranges are shown. Statistical comparisons were performed using the Kruskal-Wallis test followed by Dunn’s *post hoc* test. *p < 0.05, **p < 0.01.

Among the inflammatory cytokines, only hs-CRP was found to be significantly elevated in the PLWHIV+T2D group, with statistically significant differences compared to the PLWHIV (p = 0.043) and control groups (p = 0.009). Although no significant differences were identified for the other cytokines, a trend toward increased IL-6 levels was observed in the PLWHIV+T2D group. Additionally, a reduction in IL-18 and IL-8 concentrations was noted in the PLWHIV+preT2D group (**Figure 5**).

The discriminative ability of various surrogate indices of IR was evaluated using ROC curves, with the aim of differentiating between preT2D and T2D in PLWHIV, as well as identifying T2D in individuals without HIV. Additionally, the performance of these indices was compared between both population groups to determine in which group greater accuracy in discriminating alterations in glucose homeostasis was achieved.

**Figure 6:**
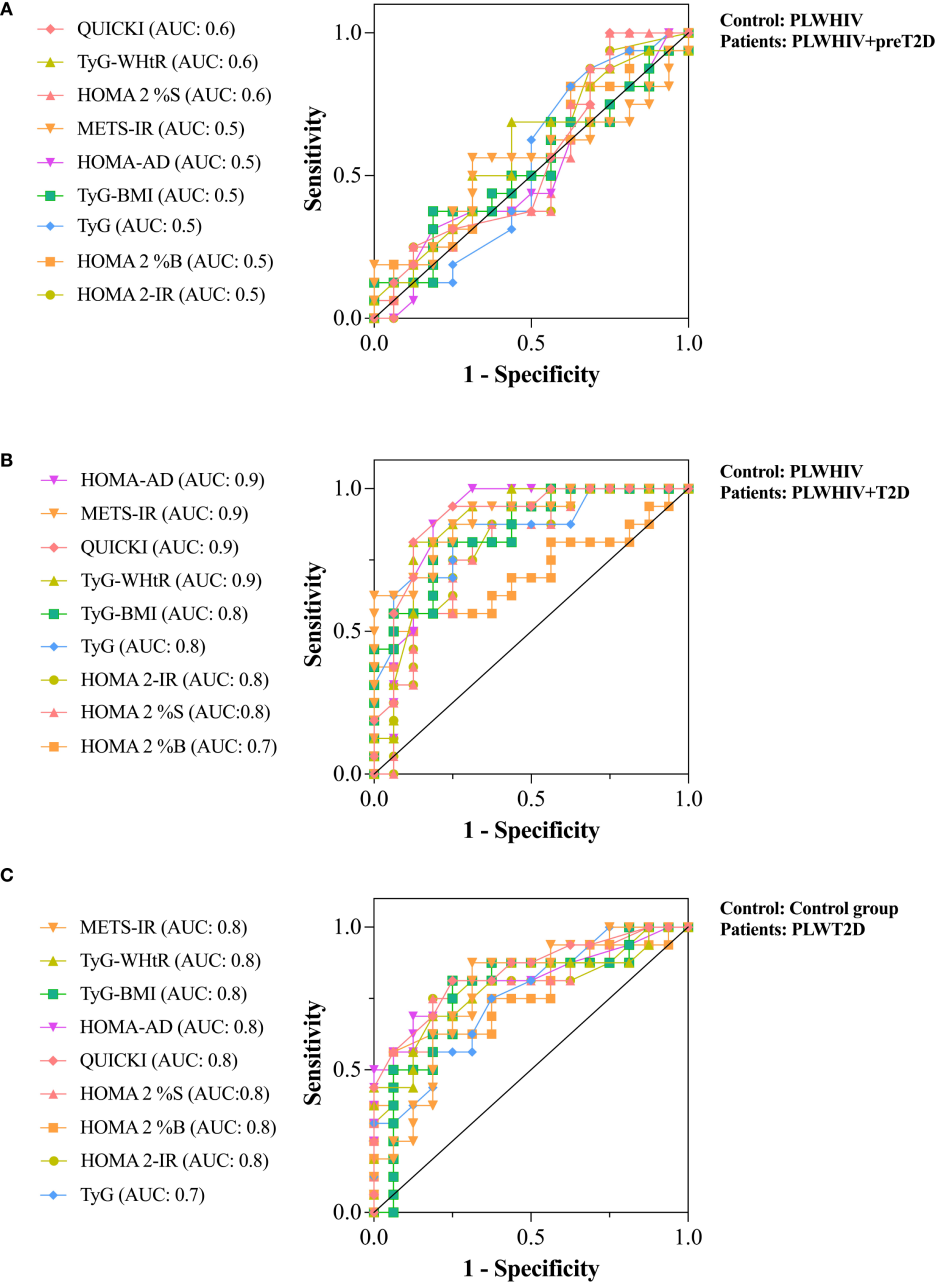
ROC curves assessing the discriminative ability of surrogate indices of IR. **(A)** Discrimination of preT2D status within the PLWHIV group. **(B)** Discrimination of T2D in the PLWHIV group. **(C)** Discrimination of T2D in individuals without HIV. Area under the curve (AUC) values are shown for each index. An AUC of 0.5 indicates no discriminative ability, 0.6–0.7 indicates poor discrimination, 0.7–0.8 moderate, and ≥0.8 excellent.

To evaluate the ability to discriminate preT2D status within the PLWHIV group, several IR indices were analyzed. The HOMA2-IR, HOMA2-%B, TyG, TyG*BMI, HOMA-AD, and METS-IR indices showed no discriminative capacity (AUC = 0.5). In contrast, HOMA2-%S, TyG-WHtR, and QUICKI exhibited poor discriminative performance, each yielding an AUC of 0.6 (**Figure 6A** **).**

For the identification of T2D in PLWHIV, HOMA2-%B showed the lowest discriminative ability (AUC = 0.7). In contrast, HOMA2-%S, HOMA2-IR, TyG, and TyG*BMI demonstrated moderate discriminative capacity (AUC = 0.8), whereas TyG-WHtR, QUICKI, METS-IR, and HOMA-AD achieved excellent discrimination (AUC = 0.9) (**Figure 6B** **).**

In the non-HIV population, none of the surrogate indices reached excellent discriminative capacity for identifying T2D. However, all evaluated indices showed moderate or acceptable performance (AUC = 0.8), except for the TyG index, which yielded an AUC of 0.7. These findings suggest that, at least in our cohort, surrogate indices of IR performed better in discriminating T2D among PLWHIV compared to those without HIV infection (**Figure 6C** **).**

**Figure 7:**
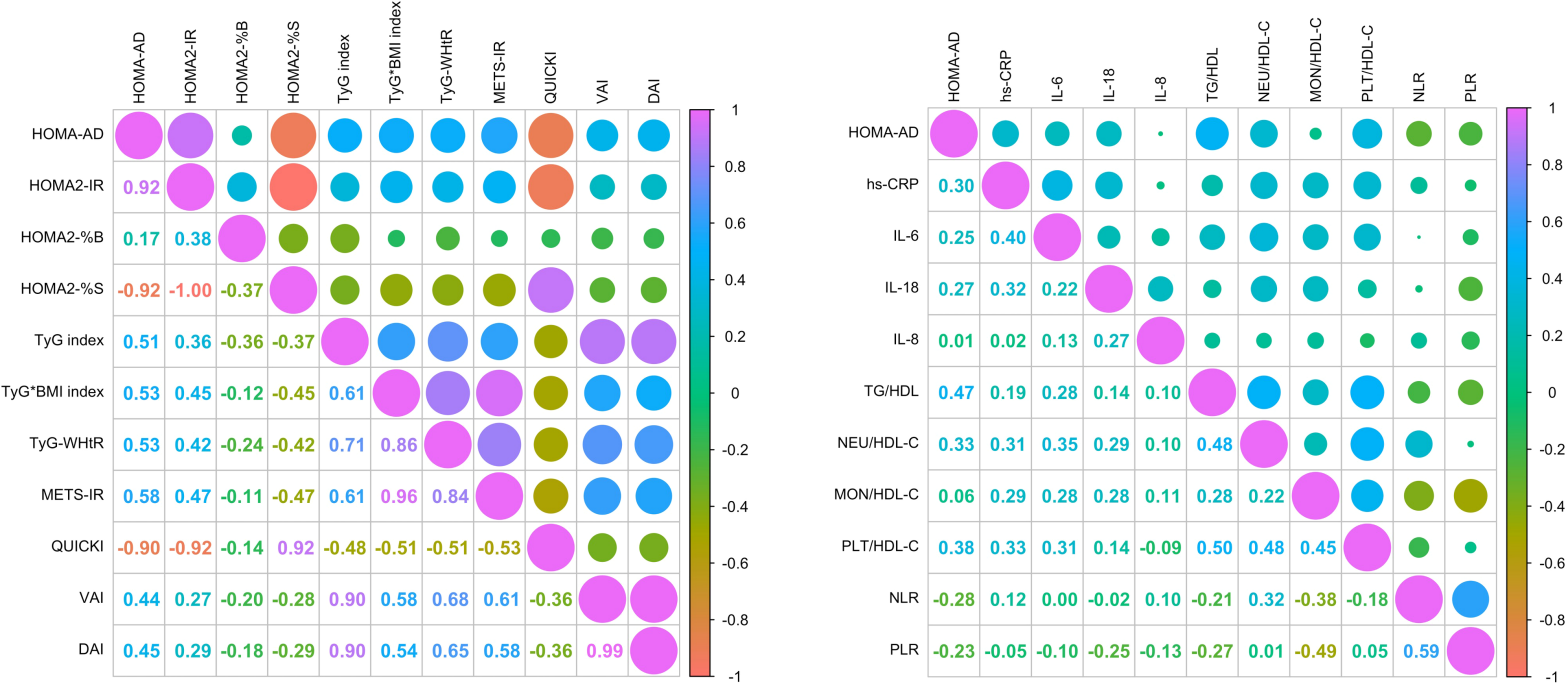
Correlation analysis between HOMA-AD, surrogate indices IR, inflammatory profile and cardiovascular risk parameters. **(A)** Correlation matrix of HOMA-AD with surrogate indices of IR and indices of visceral and dysfunctional adiposity. **(B)** Correlation matrix of HOMA-AD with inflammatory markers systemic inflammatory profile and cardiovascular risk indices. Correlations were assessed using Spearman’s correlation test.

Two correlation matrices were performed. The first aimed to identify the correlations of the HOMA-AD index with other IR indices as well as indices of visceral and dysfunctional adiposity. It was observed that METS-IR, TyG-WHtR, and QUICKI (which obtained the highest AUCs) exhibited moderate correlations with HOMA-AD: R = 0.58 for METS-IR, R = 0.53 for TyG-WHtR, and R = -0.90 for QUICKI. Nonetheless, moderate to strong correlations were shown by HOMA-AD with the remaining evaluated indices, including the adiposity indices DAI (R = 0.45) and VAI (R = 0.44) (**Figure 7A** **).**

Further, a second correlation matrix was performed to identify the correlations of HOMA-AD index with systemic inflammatory profile and cardiovascular risk. The results shown moderate correlations between HOMA-AD and cardiovascular risk and inflammatory markers, such as hs-CRP (R = 0.30), IL-6 (R = 0.25), and IL-18 (R = 0.27). Additionally, weak correlations were identified with metabolic and inflammatory indices including TG/HDL-C (R = 0.47), Neu/HDL-C (R = 0.33), PLT/HDL-C (R = 0.38), and an inverse correlation with the NLR and PLR ratios (R = -0.28 and -0.23; respectively) (**Figure 7B** **).**

**Figure 8:**
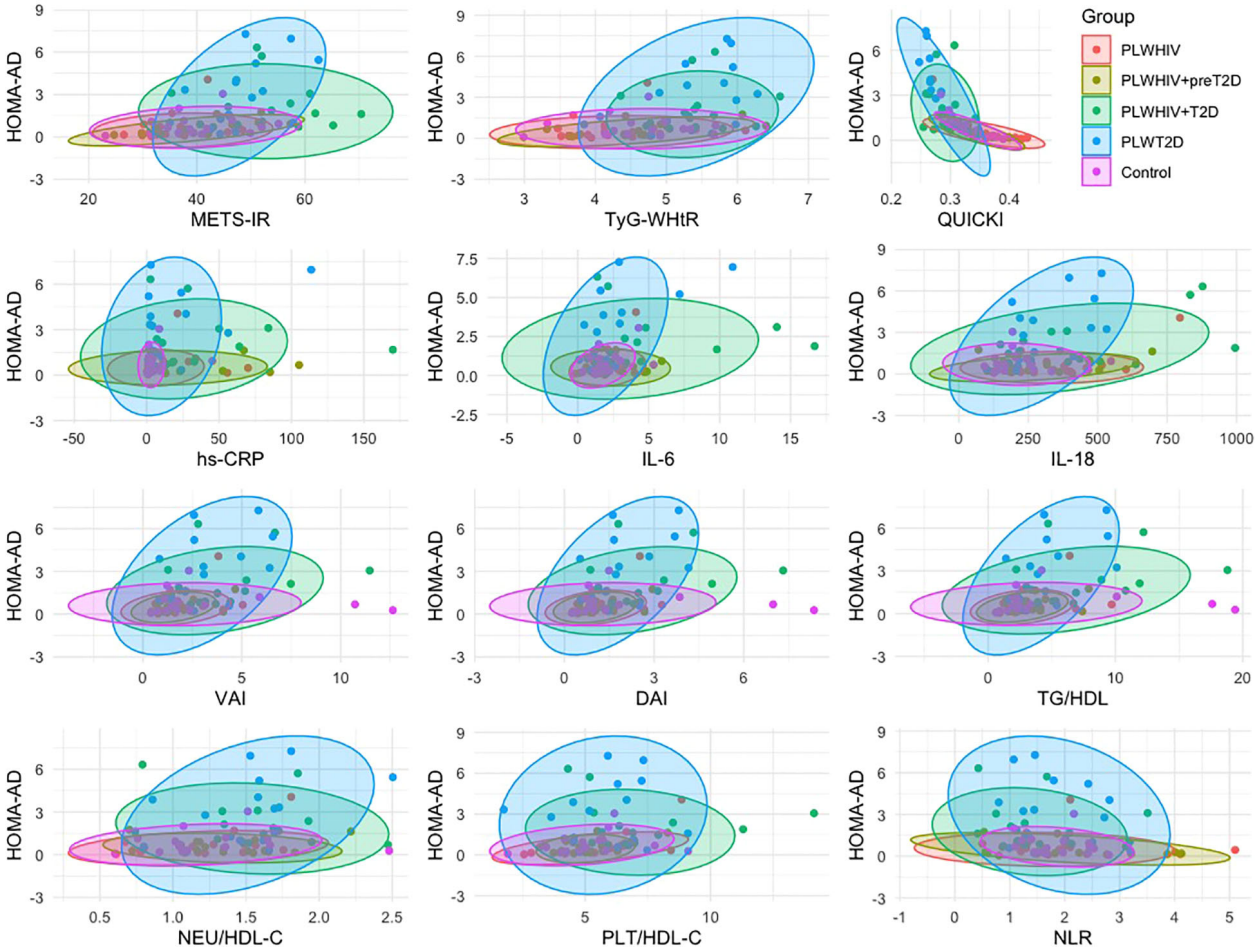
Group-specific correlations between HOMA-AD and metabolic, inflammatory, and cardiovascular risk markers. Scatter plots showing the relationship between HOMA-AD and selected surrogate indices of insulin resistance, systemic inflammatory markers, and cardiovascular risk factors, stratified by study group. Ellipses represent the 95% confidence intervals. Correlations were assessed using Spearman’s correlation test.

The associations between the HOMA-AD index and various metabolic, inflammatory, and adiposity-related parameters were explored through scatter plots stratified by group. A positive association was observed between HOMA-AD and METS-IR, TyG-WHtR, hs-CRP, IL-6, IL-18, VAI, DAI, TG/HDL, NEU/HDL-C, PLT/HDL-C, and NLR, particularly among individuals with dysglycemia (PLWHIV+preT2D, PLWHIV+T2D, and PLWT2D), in whom higher HOMA-AD values were concentrated. In contrast, an inverse correlation with QUICKI was identified, consistent with its interpretation as an insulin sensitivity index. The density ellipses suggested a clear separation between normoglycemic individuals and those with metabolic alterations, with a more pronounced dispersion observed in the T2D groups, regardless of HIV serostatus (**Figure 8** **).**

**Figure 9:**
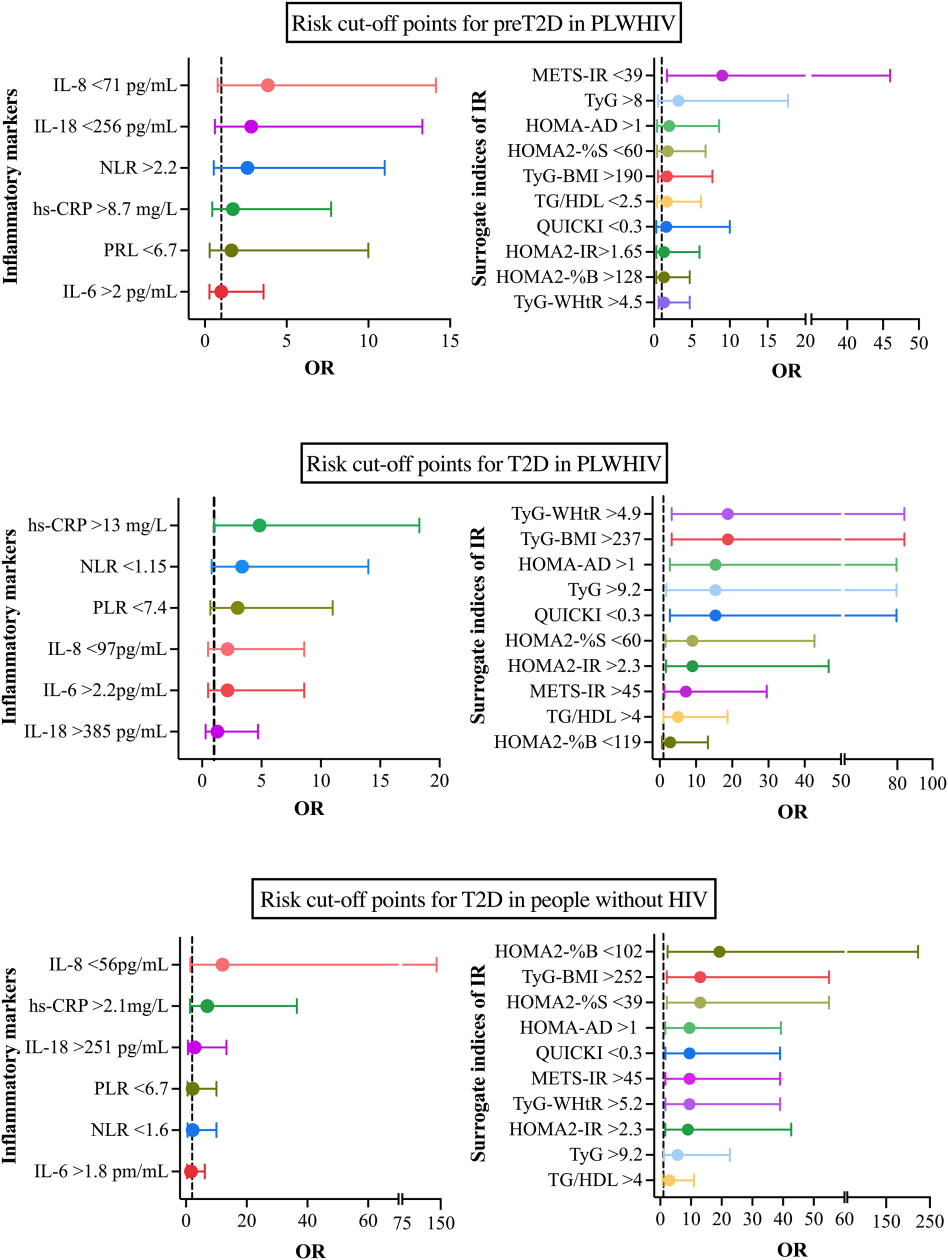
Cut-off points associated with the risk of preT2D and T2D in people with and without HIV. **(A)** Cut-off points most strongly associated with preT2D risk in PLWHIV. **(B)** cut-off points associated with T2D risk in PLWHIV. **(C)** cut-off points for T2D risk in people without HIV. Odds ratios (OR) and 95% confidence intervals (95% CI) are shown for each inflammatory marker (left) and IR marker (right). Cut-off points were determined using ROC curve analyses and are indicated alongside each variable.

Based on the analysis of the ROC curves, the optimal cutoff points were determined by selecting those with the highest sensitivity and specificity, in addition to considering the highest Youden index and the best Euclidean distance. Based on these values, subgroups were established within each study group, with participants being classified according to whether their values were above or below the established cutoff points. Subsequently, contingency tables were constructed for each subgroup with the aim of calculating the odds ratios (OR) and 95% confidence intervals (95% CI), thereby permitting the evaluation of the risk associated with each cutoff point. The obtained results were represented using forest plots for each of the established comparisons (**Figure 9**).

In the population of individuals with PLWHIV+preT2D, the primary cutoff points identified were HOMA-AD >1 (OR = 1.97, 95% CI: 0.35–8.6), NLR >2.2 (OR = 2.6, 95% CI: 0.54–11), PRL<6.7 (OR = 1.615, 95% CI: 0.2873–10), hs-CRP >8.7 (OR = 1.711, 95% CI: 0.4527–7.722), IL-6 >2 (OR = 1, 95% CI: 0.2789–3.585), IL-18 <256 (OR = 2.829, 95% CI: 0.6048–13.3), and IL-8 <71 (OR = 3.857, 95% CI: 0.7742–14.13). In the group of individuals with PVVIH+T2D, the most relevant cutoff points were identified as HOMA-AD >1 (OR = 15.4, 95% CI: 2.787–79.5), NLR <1.15 (OR = 3.37, 95% CI: 0.8–14), PLR <7.4 (OR = 3, 95% CI: 0.7–11), hs-CRP >13 (OR = 4.84, 95% CI: 1–18.3), IL-6 >2.2 (OR = 2.143, 95% CI: 0.5–8.6), IL-18 >385 (OR = 1.286, 95% CI: 0.3–4.7), and IL-8 <97 (OR = 2.143, 95% CI: 0.5–8.6). Finally, in the population without HIV but with a diagnosis of T2D, the identified cutoff points were HOMA-AD >1 (OR = 9.533, 95% CI: 1.653–39.3), NLR <1.6 (OR = 2.2, 95% CI: 0.5356–10), PLR <6.7 (OR = 2.2, 95% CI: 0.5356–10), hs-CRP >2.1 (OR = 7, 95% CI: 1.329–36.5), IL-6 >1.8 (OR = 1.653, 95% CI: 0.3658–6.2), IL-18 >251 (OR = 2.829, 95% CI: 0.6048–13.3), and IL-8 <56 (OR = 12, 95% CI: 1.419–142.2).

The specific values of the cutoff points, along with their respective OR and 95% CI, are presented in the corresponding figures, thereby providing a visual representation of the magnitude of risk in each scenario.

## 4 Discussion

Chronic inflammation and AT dysfunction have been identified as key contributors to the development of an adverse immunometabolic state. However, this phenomenon has been insufficiently characterized in PLWHIV, due to the complex interplay among persistent immune activation, the metabolic side effects of ART, and HIV-induced AT redistribution. Since the introduction of ART, HIV infection has been transformed from a fatal disease into a chronic condition associated with prolonged life expectancy. As a result, a reduction in opportunistic infections has been observed, along with an increased prevalence of NCDs, including MetS, T2D, and cardiovascular disease (29). This elevated risk has been attributed not only to traditional factors, but also to chronic inflammation, ART-related metabolic disturbances, and weight gain (30). Increased AT has been associated with elevated levels of proinflammatory mediators and free fatty acids, both of which have been implicated in the development of IR (31, 32), a condition that often precedes the onset of T2D (33). This study is intended to evaluate the HOMA-AD index, a marker of RI and metabolic risk, and its correlation with metabolic, inflammatory parameters obesity-related.

Metabolic disturbances have been reported to emerge at younger ages in PLWHIV compared to the general population (34). Our findings support the observation that PLWHIV tend to debut earlier with preDT2 or DT2 diagnostic than individuals without HIV, suggesting that HIV infection and its treatment may contribute to the earlier onset of glucose metabolism alterations. This difference, along with other reported studies to date, suggests that in the context of HIV, metabolic dysfunction may develop more rapidly, possibly driven by mechanisms such as chronic immune activation, premature immunosenescence, and AT dysfunction induced either by the infection itself or by ART (35).

Consistent with the accelerated progression of metabolic dysfunction, significant differences in body composition were observed among the groups. PLWHIV with T2D exhibited greater visceral fat accumulation, reflecting a more compromised anthropometric profile that may contribute to an increased risk of metabolic complications. Consistent increases in these parameters have been previously reported in this population, reinforcing concerns about a tendency towards lipohypertrophy and visceral fat deposition (36), a phenomenon closely associated with chronic inflammation, IR, and elevated cardiovascular risk (37).

In contrast, the control group exhibited a general pattern of overweight, a risk for metabolic disturbances. This finding is consistent with national reports on the nutritional status of Mexican adults, which demonstrate a high prevalence of undiagnosed overweight and obesity in the mexican general population (38). Notably, PLWHIV without glycemic alterations exhibited a lower body mass pattern, which may reflect stricter clinical management or differences in body fat distribution associated with HIV.

WHR progressively increased in accordance with the degree of glycemic impairment, reaching its highest value in the PLWHIV+T2D group. Transcendentally, WHR has been shown to be a more accurate predictor of cardiometabolic risk than BMI in PLWHIV (39). In this context, ART particularly regimens containing INSTIs such as bictegravir combined with tenofovir alafenamide has been associated with significant weight gain and increased risk of obesity (40, 41). It has been documented that the use of PIs, NRTIs, and NNRTIs is linked to weight gain, obesity, and metabolic dysfunction through several molecular mechanisms. PIs can block GLUT-4 transporters, impair adipocyte differentiation, and activate inflammatory pathways such as NF-κB, promoting IR and visceral adiposity. Older NRTIs induce mitochondrial DNA depletion and dysfunction, leading to impaired oxidative phosphorylation, adipocyte apoptosis, and lipodystrophy. Finally NNRTIs, may alter hepatic lipid metabolism through cytochrome P450 interactions, contributing to dyslipidemia (29). Particularly, INSTIs use modulate central appetite regulation through interactions with melanocortin and melanocortin-4 receptors, alterations in adipocyte differentiation and lipid storage, as well as potential effects on mitochondrial function, and intestinal dysbiosis, thus affecting IR and increase of fat mass in the upper body (29, 42, 43). Recently, it was reported an T2D incidence rate of 24.6/1000 person-years in PLWHIV with INSTIs, additionally the cumulative incidence of T2D was 6.7% and 11.7% at 3 and 5 years of receiving ART based in INSTIs (44). In our cohort, most participants were on the same antiretroviral regimen, minimizing pharmacological variability. Furthermore, PLWHIV exhibited stable clinical and immunological control, as evidenced by viral suppression and adequate CD4^+^ cell counts.

Clinical characterization of the groups revealed that both HIV diagnosis duration and ART exposure were longer in participants with glycemic alterations, suggesting a potential link between infection chronicity and metabolic dysfunction. This finding is particularly relevant, as both prolonged HIV infection and extended ART exposure have been associated with abnormal adipose tissue distribution, low-grade chronic inflammation, mitochondrial dysfunction, and progressive increases in insulin resistance, even in individuals maintaining effective virological suppression (45).

Furthermore, although differences in CD4^+^ nadir were not statistically significant, a trend toward higher values was observed in the PLWHIV+T2D group, potentially reflecting a more robust immune reconstitution. However, immune reconstitution does not imply reversal of the underlying metabolic damage; rather, recent studies have suggested that even with an apparently restored immunological profile, a residual inflammatory milieu persists, capable of impairing insulin signaling in peripheral tissues, particularly adipose tissue (46). These findings emphasize the need to assess HIV-specific factors, including diagnosis duration, ART exposure, and immune reconstitution. Despite virological control and adequate CD4^+^ counts, HIV and prolonged ART are linked to AT changes, inflammation, IR, and increased metabolic risk.

Consistently, the PLWHIV+T2D group exhibited markedly reduced HDL-C levels, a profile associated with inflammation, IR, and increased cardiometabolic risk (47). Fasting glucose and HbA1c levels were elevated in PLWHIV groups, in line with studies reporting early glycemic abnormalities in this population, even prior to a formal T2D diagnosis (48). These findings support the notion that metabolic dysfunction in PLWHIV may develop early and in a subclinical manner. Furthermore, the group with the lowest HDL-C concentration (PLWHIV+T2D) also exhibited reduced AD levels, a pattern reported previously (47, 49) and consistently confirmed in recent studies (50).

Adiponectin levels may increase during the early stages of glycemic dysfunction, such as in preT2D, functioning as a compensatory mechanism to enhance insulin sensitivity. However, as the condition progresses toward established T2D, its levels tend to decline, reflecting a progressive deterioration of adipose tissue function (51). This pattern was consistent with our observations.

PLWHIV+T2D exhibited a pronounced inflammatory profile, evidenced by elevated hs-CRP and IL-18 levels, along with a trend toward increased IL-6, reflecting an exacerbated proinflammatory state. These alterations are consistent with recent studies linking persistent low-grade inflammation in PLWHIV to increased cardiometabolic risk and progression to T2D (52, 53). Collectively, these findings underscore the relevance of integrating inflammatory and adipokine biomarkers to better characterize the immunometabolic profile in this population. Elevated hs-CRP levels have been associated with a cyclical cascade of low-grade chronic inflammation in response to the release of pro-inflammatory cytokines such as IL-6 and TNF-α (54); this is positively correlated with IR and represents a risk factor for the development of T2D (55).

In this context, composite indices integrating metabolic, adipokine related, and anthropometric parameters such as HOMA2-IR, HOMA-AD, TyG, METS-IR, VAI, and DAI represent valuable tools for detecting early IR and metabolic disturbances in PLWHIV, even prior to a formal T2D diagnosis. Their application is particularly relevant in populations such as PLWHIV, where glycemic dysfunction tends to manifest at younger ages, highlighting the need for more timely screening strategies.

The gold standard for measuring IR euglycemic hyperinsulinemic clamp; however, given its complexity, several surrogate indices have been developed and validated, among them the most common is the HOMA-IR (56) In our study, significant differences were observed in indices related to IR and AT dysfunction among the groups.

The PLWHIV+T2D group exhibited severe IR accompanied by impaired pancreatic beta-cell function. This pattern reflects advanced metabolic dysfunction, characterized by a dual defect of IR and inadequate insulin secretion hallmarks of a complex diabetic phenotype. Reduced HOMA-%B values have been reported in PLWHIV receiving ART, indicating impaired β-cell function potentially related to immune reconstitution (46, 57). Moreover, historically the Ips has been related to alterations to glucose and lipid metabolism (58); however also it has been suggested that the use of INSTI could be related to an accelerated dysglycemic process and contribute to beta-cell dysfunction and IR independent of weight gain (59). In HIV-associated lipodystrophy, decreased insulin sensitivity and clearance, despite preserved secretion, suggest limited β-cell compensation and worsening metabolic dysfunction (60).

HOMA-AD has been proposed as a superior index for assessing IR by incorporating adiponectin, a key marker of adipose tissue function (20). Given the frequent adipose dysfunction in PLWHIV, this index may better capture their metabolic disturbances. In our study, HOMA-AD was significantly elevated in the PLWHIV+T2D group, supporting its potential clinical utility. On the other hand, in PLWHIV+preT2D, we observed the lowest HOMA-AD values. Interestingly, as shown in the previous results, this group exhibited the highest levels of insulin and adiponectin, which may reflect a compensatory response to hyperglycemia and persistent metabolic inflammation. Since both variables are included in the calculation of HOMA-AD, their elevation is associated with lower index values in PLWHIV+preT2D. This finding suggests that HOMA-AD may not be the most accurate tool for identifying IR in this group and could potentially underestimate its presence an intriguing hypothesis that warrants further investigation.

This study provides the first evidence of HOMA-AD application in PLWHIV, offering a novel approach to assess IR by incorporating adiponectin-mediated AT dysfunction, a frequently overlooked component in HIV-associated metabolic alterations. Although our sample size could be considered a limitation for our study, it is important to highlight that other clinical studies have been published in PLWHIV and metabolic conditions, which strengthens the findings of this study (61, 62). Studies have confirmed that HOMA-AD shows a stronger correlation with IR than HOMA-IR, demonstrating superior diagnostic performance in adults, particularly when compared to the hyperglycemic clamp method (22).

In this study, the metabolic indices TyG, TyG*BMI, TyG-WHtR, METS-IR, and QUICKI showed significant differences among the analyzed groups. Elevated TyG values in PLWHIV+T2D and PLWT2D indicate increased IR, consistent with previous reports in patients with T2D. Indices that incorporate adiposity, such as TyG*BMI and TyG-WHtR, were also higher in these groups, reflecting the contribution of general and central adiposity to metabolic impairment.

METS-IR, a composite marker integrating lipid and anthropometric parameters, showed higher values in PLWHIV+T2D and PLWT2D, confirming increased IR in these groups. Conversely, QUICKI, which is inversely related to IR, was lower in the same groups, indicating reduced insulin sensitivity.

These findings highlight that the coexistence of HIV and T2D is associated with an unfavorable metabolic profile characterized by IR and central adiposity factors that increase cardiovascular risk and metabolic complications in this population.

VAI and DAI were elevated in PLWHIV+T2D, suggesting greater visceral fat accumulation and AT dysfunction. This group also exhibited an altered inflammatory profile, characterized by lower HDL-C and AD levels, higher HbA1c and IL-6, as well as a reduced beta-cell function. These findings highlight the critical role of AT as a modulator of the metabolic and inflammatory milieu, potentially exacerbated by chronic HIV infection and prolonged ART. Previous studies have validated VAI as an indirect marker of IR and adipose dysfunction, while elevated DAI values have been associated with metabolic conditions including T2D, hepatic steatosis, and atherosclerosis, supporting their utility in cardiometabolic risk assessment in PLWHIV (63, 64).

Several indices related to blood components and HDL-C, including TG/HDL-C, NEU/HDL-C, MON/HDL-C, PLT/HDL-C, as well as NLR and PLR, were assessed. Among these, TG/HDL-C, MON/HDL-C, and PLT/HDL-C differed significantly between groups. The elevation of TG/HDL-C in PLWHIV+T2D and PLWT2D reflects an atherogenic lipid profile strongly associated with IR, subclinical inflammation, and increased cardiometabolic risk, consistent with previous reports (65).

Likewise, the higher MON/HDL-C ratio observed in PLWHIV+T2D aligns with its proposed role as a surrogate inflammatory marker in predicting endothelial dysfunction and cardiometabolic complications (66). These findings highlight the importance of assessing not only traditional metabolic parameters but also inflammatory and cellular markers related to immune and coagulation systems to better understand cardiovascular risk in HIV population.

Based on these findings, only the indices that showed significant differences between groups and well-defined distributions were selected for discriminative capacity analysis. In PLWHIV+preT2D, all surrogate indices demonstrated limited ability to detect early alterations in glucose metabolism, suggesting reduced clinical utility at this stage (67).

In contrast, PLWHIV+T2D, certain composite indices exhibited excellent discriminative performance, aligning with previous studies identifying IR and hypertriglyceridemia as robust predictors of T2D in PLWHIV. The incorporation of adiponectin into HOMA-AD enhances its clinical relevance under chronic inflammation. Among PLWT2D, most indices performed satisfactorily, highlighting the value of composite indices integrating biochemical and anthropometric parameters. Their consistent performance in non-HIV populations underscores their cost-effective potential for metabolic and cardiometabolic risk screening (68, 69).

To our knowledge, this is one of the first studies to comprehensively evaluate both traditional and surrogate IR indices in a cohort with varying degrees of metabolic alterations and HIV infection. Furthermore, we demonstrate that these indices perform better in the PLWHIV compared to HIV-negative individuals, as evidenced by the ROC curve analysis, highlighting their clinical utility for detecting metabolic complications in this specific group.

HOMA-AD represents a useful tool for assessing IR and dysfunctional visceral adiposity. In the present study, it demonstrated moderate associations with surrogate indices of IR and adiposity, including TyG, TyG*BMI, TyG-WHtR, and METS-IR, supporting its clinical relevance. However, indices such as TyG and METS-IR showed more consistent associations with measures of dysfunctional adiposity (VAI and DAI), indicating that they may be particularly valuable for practical and effective characterization of altered adiposity profiles. These findings are consistent with previous studies showing superior performance of HOMA-AD in detecting IR, while METS-IR relates more closely to MetS components (26). HOMA-AD strongly correlates with HOMA-IR and better reflects IR prevalence, supporting its use in populations with metabolic alterations (70). The association between visceral fat to skeletal muscle ratio and multiple IR indices, including HOMA-AD, highlights the importance of body composition in cardiometabolic risk (71).

In our study, HOMA-AD was moderately associated with inflammatory and metabolic markers, including hs-CRP and ratios (TG/HDL-C, NEU/HDL-C and PLT/HDL-C), while weaker associations were observed with proinflammatory cytokines such as IL-6 and IL-18. These findings indicate that HOMA-AD reflects both IR and subclinical inflammatory status. Previous reports also linked HOMA-AD with inflammatory biomarkers, including PAI-1 in severe obesity in brazilian population (72). Additionally in caucasic population, significant associations between HOMA-AD, adiponectin, body composition, and diet emphasize its utility to evaluate IR and inflammatory status in metabolic disorders as well as to improve lifestyle. However, it is important to consider that body distribution and diet vary between populations (73).

Our study found that in PLWHIV and dysglycemia, HOMA-AD showed positive associations with METS-IR, TyG-WHtR, hs-CRP, IL-6, IL-18, VAI, and DAI, reflecting IR, chronic inflammation, and dysfunctional visceral adiposity. Its inverse correlation with QUICKI supports its validity as an insulin sensitivity marker. The clear separation in density plots between normoglycemic and metabolically altered individuals suggests that HOMA-AD effectively discriminates metabolic states in PLWHIV regardless of glucemic status. These findings indicate that HOMA-AD is a promising comprehensive index for assessing metabolic risk and inflammation in this vulnerable population. Importantly, to our knowledge, HOMA-AD has not been previously evaluated in people living with HIV, positioning our study as a novel contribution to understanding its applicability in this specific clinical context.

Finally, our study identified cutoff points for various biomarkers that reflect the risk of T2D in populations with and without HIV. Notably, HOMA-AD >1 proved particularly sensitive in identifying elevated risk among PLWHIV+T2D. Other indices, including QUICKI, TyG, TyG*BMI, and TyG-WHtR, also demonstrated significant utility, highlighting the advantage of integrating biochemical and anthropometric parameters for a more comprehensive assessment of IR and cardiometabolic risk in vulnerable populations. These findings support the efficacy of HOMA-AD, comparable to HOMA-IR and TyG, in predicting MetS, central obesity, and dyslipidemia conditions that precede T2D (25). Moreover, its stronger correlation with other indices and its integrative capacity combining biochemical and anthropometric parameters make it a key marker for assessing and managing metabolic risk in PLWHIV.

There is limited research evaluating cutoff points and odds ratios of IR indices in general population and even more in PLWHIV. However, some reports have shown that in PLWHIV, the TyG index has a strong correlation with HOMA-IR (r = 0.628) and a cutoff point of 8.25 for identifying IR (74), consistent with our findings and confirming its clinical utility. In addition, among HIV patients receiving ART in Indonesia, the HOMA-IR cutoff point was 2.705, with 70% sensitivity and specificity (75), supporting the need for population-specific thresholds to enhance early detection of IR in this group.

A limitation of our study is the small sample size and the cross-sectional design, which precludes causal inferences. Future longitudinal studies with larger cohorts should confirm these findings and include multivariate analyses to adjust for potential confounders such as lifestyle, medications, and comorbidities. Furthermore, another limitation is the lack of data from other populations with HIV and T2D to be able to extrapolate our results, because this is the first study to evaluate HOMA-AD in this context.

## 5 Conclusions

Our findings highlight a strong association between HOMA-AD, surrogate indices of IR, and markers of inflammation with the T2D in PLWHIV. In particular, PLWHIV with T2D showed higher HOMA-AD, HOMA2-IR, TyG, and METS-IR values, along with lower AD and elevated hs-CRP and IL-18 levels, reflecting a pronounced state of IR and systemic inflammation. These data support the utility of HOMA-AD and related indices as accessible tools for early detection and cardiometabolic risk stratification in this population.

## Data Availability

The original contributions presented in the study are included in the article/supplementary material. Further inquiries can be directed to the corresponding author.
